# Impact of Different Dosage Forms of Orally Administered Chinese Herbal Medicine on Treatment and Adverse Effect Estimates in Randomized Controlled Trials: A Meta‐Epidemiological Study

**DOI:** 10.1111/jebm.70115

**Published:** 2026-01-31

**Authors:** Claire Chenwen Zhong, Betty Huan Wang, Mary Yue Jiang, Leonard Ho, Fai Fai Ho, Irene Xin Yin Wu, Yin Ting Cheung, Vincent Chi Ho Chung

**Affiliations:** ^1^ The Jockey Club School of Public Health and Primary Care Faculty of Medicine The Chinese University of Hong Kong Hong Kong China; ^2^ School of Chinese Medicine Faculty of Medicine The Chinese University of Hong Kong Shatin Hong Kong China; ^3^ Xiangya School of Public Health Central South University Changsha Hunan China; ^4^ School of Pharmacy Faculty of Medicine The Chinese University of Hong Kong Shatin Hong Kong China

**Keywords:** chinese herbal medicine, chinese traditional medicine, dosage forms, drugs, meta‐epidemiological study

## Abstract

**Aim:**

In traditional Chinese medicine, different dosage forms of orally administered Chinese herbal medicine (CHM) may introduce bias in estimating treatment and adverse effects. This meta‐epidemiological study aimed to evaluate whether the use of different orally administered CHM dosage forms is associated with overestimation or underestimation of treatment and adverse effects in randomized controlled trials (RCTs).

**Methods:**

Seven electronic databases were searched to identify potentially eligible meta‐analyses (MAs) of RCTs evaluating CHM interventions. A two‐step meta‐epidemiological analysis was performed, using ratios of odds ratios for binary outcomes and differences in standardized mean differences for continuous outcomes. These metrics assessed whether different orally administered CHM dosage forms—including CHM decoctions, Chinese patent medicines (CPMs), and CHM granules influenced the magnitude of reported treatment effects or adverse effects.

**Results:**

Eighty‐two MAs comprising 1263 RCTs were analyzed. Overall, there was no consistent evidence that any oral dosage form systematically overestimated or underestimated treatment effects or adverse effects. Sensitivity analyses confirmed these findings, with the exception that CHM decoctions showed slightly larger binary treatment effects compared to CPMs after adjusting for incomplete outcome data. However, when adjusted for all confounders, CPMs yielded significantly greater continuous treatment effects than CHM decoctions. Additionally, CHM granules were associated with larger continuous treatment effects than CHM decoctions after adjusting for RCT funding. Subgroup analyses indicated that RCTs on digestive diseases tended to report larger effect estimates when using CHM decoctions, whereas RCTs on endocrine, nutritional, and metabolic diseases tended to report larger effect estimates when using CPMs.

**Conclusions:**

This meta‐epidemiological study suggests that while oral dosage forms of CHM are associated with minimal differences in reported treatment and adverse effect estimates, specific dosage forms may offer advantages in certain contexts. Subgroup analyses indicate that digestive disease trials tend to report larger estimates with CHM decoctions, and endocrine/metabolic disease trials with CPMs. When adjusting for confounders, CPMs yield greater continuous treatment effects compared with CHM decoctions, while CHM granules are associated with larger estimates than CHM decoctions after adjusting for RCT funding. Further research is needed to confirm their clinical relevance and guide formulation choices in CHM practice.

## 1 Introduction

Traditional Chinese medicine (TCM) is a holistic medical practice with unique diagnostic methods and therapeutic principles that originated in China and is widely used as a type of complementary medicine in many Western countries [1]. A fundamental component of TCM is Chinese herbal medicine (CHM), which has been employed for thousands of years to enhance health and treat various conditions [1]. Traditionally, CHM has been administered in the form of decoctions, where raw herbs are boiled to extract their active ingredients [2]. The composition of these herbal materials within a decoction can be tailored according to the patient's condition, as determined through TCM syndrome differentiation. Despite their longstanding use and therapeutic relevance, decoctions pose several challenges, including difficulties in ensuring consistent quality of herbal ingredients, time‐consuming preparation, practical issues relating to transportation and storage, and the requirement to ingest large volumes of often unpleasant‐tasting medicine [2, 3]. These factors can lead to decreased patient adherence and may compromise the therapeutic effects of CHM [4].

To address these challenges, the development of CHM granules has emerged as a significant advancement. CHM granules are a modern form of TCM formulation that offers convenience and standardization in dosing [5]. These granules are produced by extracting, concentrating, drying, and granulating herbal materials. Patients or TCM practitioners can easily mix and dissolve these granules in hot water to create a decoction, allowing for accurate dosing and simpler administration [4]. Due to their portability, ease of preparation, and consistent quality [6, 7], CHM granules have become widely accepted, not only in China but also in other Asian countries such as South Korea and Japan, and are gradually being recognized by Western pharmaceutical markets, including those in the United Kingdom, the United States, and Germany [8]. In China, over 700 types of herbal formula granules are utilized in clinical practice, with annual sales reaching billions of dollars [4, 9].

Chinese patent medicines (CPMs) are another form of CHM, approved by the National Drug Regulatory Authority of China and manufactured according to standardized procedures using Chinese herbs as raw materials, following TCM theory [10]. These medicines are typically available in forms such as pills, tablets, capsules, or injections, providing a convenient method for CHM administration [11]. The use of CPMs should also be guided by the principles of syndrome differentiation and comprehensive analysis of signs and symptoms [10]. In China, 1617 CPMs are included in the *Pharmacopoeia of the People's Republic of China (2020 Edition)* [12]. Compared to CHM decoctions and granules, CPMs offer greater convenience, portability, and ease of use, making them prevalent in clinical practice [10]. However, CPMs lack the adaptability of decoctions and granules, which can be customized to meet the evolving needs of individual patients based on TCM syndrome differentiation. This customization is closely aligned with TCM theory and is particularly beneficial in clinical scenarios where patient conditions may vary.

As TCM increasingly incorporates evidence‐based approaches, the comparative effectiveness and safety of different orally administered CHM dosage forms have become important areas of research [13]. Questions arise regarding how granules and CPMs compare to traditional decoctions in terms of treatment and adverse effects [14]. Additionally, there is ongoing debate over whether granules can serve as a suitable replacement for traditional decoctions [4, 14]. Both producers and consumers of CHM frequently encounter confusion and uncertainty about these issues. Previous systematic reviews (SRs)that have compared the effectiveness and safety of granules with decoctions have not provided conclusive evidence on whether CHM granules offer equivalent effectiveness and safety in clinical settings [2, 4]. Furthermore, there is a lack of comparative data on the effectiveness and safety of these three orally administered CHM dosage forms.

Meta‐epidemiological studies, which have gained prominence over the past two decades, analyze how specific characteristics of clinical trials influence observed outcomes [15]. By comparing effect estimates between trials with and without certain characteristics of interest, these studies employ SR or meta‐analysis (MA) approaches to examine the impact of these features on observed effects, providing empirical evidence for hypothesized associations [15, 16]. Therefore, by conducting a meta‐epidemiological analysis of SRs and randomized controlled trials (RCTs), this study aims to systematically assess whether different orally administered CHM dosage forms, including CHM decoctions, CPMs, and CHM granules, are associated with overestimation or underestimation of treatment effects or adverse effects in CHM RCTs. In this study, CPMs were grouped based on their shared characteristics: they are standardized, industrially manufactured products with fixed formulations approved by regulatory authorities. Decoctions are freshly prepared and highly flexible in composition, allowing for real‐time adjustments according to clinical presentation. Granules, while standardized in extraction, still retain some flexibility in combining formulas for individualized use. This distinguishes them from decoctions and granules, which are typically tailored to individual patients based on syndrome differentiation. Such categorization aimed to reflect these practical and clinical distinctions rather than pharmaceutical formats. By undertaking this comprehensive evaluation, we seek to clarify the impact of orally administered dosage forms on therapeutic outcomes and identify potential areas for further investigation and refinement in CHM practice.

## 2 Methods

We conducted this meta‐epidemiological study based on the guidelines for reporting meta‐epidemiological research methodology [15]. We registered the protocol in PROSPERO (*No*. CRD42024552112) [17].

### 2.1 Eligibility Criteria

Eligible SRs in this study are required to include at least one eligible MA pooling RCTs evaluating the treatment or adverse effects of CHM with various orally administered CHM dosage forms. Eligible orally administered CHM dosage forms include CHM decoctions, CPMs, and CHM granules. We performed meta‐epidemiological analysis to evaluate the differences in effect estimates between the following pairwise comparisons: RCTs using CHM decoctions vs. RCTs using CHM granules, RCTs using CHM decoctions vs. RCTs using CPMs, and RCTs using CPMs vs. RCTs using CHM granules. To ensure statistical robustness, eligible MAs should include a minimum of ten eligible RCTs to allow for the evaluation of at least one of the above comparisons [18]. MAs involving RCTs with the same dosage form of orally administered CHMs were excluded.

Only the MA with the most RCTs were analyzed when multiple eligible MAs are available in the same SR [19]. We also excluded overlapping RCTs from the included MAs. After this process, MAs with fewer than ten eligible RCTs were excluded, ensuring a non‐overlapping dataset [20].

No restrictions were applied regarding patient characteristics, language, or disease of interest to the RCTs included in the MAs. However, interventions, comparisons, and outcomes had to meet specific criteria. (a) Interventions and comparisons: Interventions had to be orally administered CHMs of any eligible dosage form, with at least one ingredient from the *Pharmacopoeia of the People's Republic of China (2020 Edition)* [12]. RCTs evaluating other TCM modalities like acupuncture and therapeutic massage (Tuina) were excluded. RCTs assessing the following comparisons were considered eligible, including (i) CHM vs. conventional treatment; (ii) CHM in conjunction with conventional treatment vs. conventional treatment; and (iii) CHM vs. placebo. Conventional treatment refers to the medical therapies that is widely accepted and used by most health care professionals [21], encompassing Western medicine, biomedicine, scientific medicine, and modern medicine [22]. Each MA had to include consistent comparisons across RCTs. Only the two arms consistent with other RCTs in the same MA were included in the analysis. (b) Outcomes: We included all RCTs reporting treatment effects or adverse effects, irrespective of whether these were classified as binary or continuous outcomes.

We excluded network MAs, overviews of SRs, RCT protocols, abstracts, animal studies, and RCTs without full text. The latest version of SRs was included in this study when multiple versions were available.

### 2.2 Literature Search

We searched MEDLINE, Embase, Cochrane Database of SRs, China National Knowledge Infrastructure (CNKI), WanFang, Chinese Biomedical Literature Database, and Airiti Library for potential eligible studies published from 2021 to September 15, 2022. We adopted maximized specialized filters for SRs in MEDLINE and Embase [23]. The search strategies were shown in Supplementary Material A.

### 2.3 Literature Screening

The EndNote 20 was used to remove duplicated studies for all citations searched and imported. Following this, we screened titles and abstracts against our eligibility criteria, and further assessed full texts to select eligible SRs and MAs. After identifying a list of potential SRs and MAs, we evaluated the eligibility of RCTs embedded within these eligible MAs.

Before commencing the study, reviewers underwent extensive training and calibration to ensure a robust inter‐rater agreement. A reviewer independently screened potentially eligible studies, extracted data, and assessed the risk of bias (RoB) of screened studies. To ensure reliability, a second reviewer independently replicated these steps for a randomly selected subset comprising one‐fifth of the trials. Discrepancies were resolved through discussion by both reviewers. In cases where consensus could not be reached, a third reviewer was consulted for adjudication.

### 2.4 Data Extraction

We used a pre‐developed data extraction form (Supplementary Material B) to capture the general characteristics of MAs and embedded RCTs. We gathered all data on treatment effects and adverse effects, irrespective of whether they were binary or continuous. These outcomes were then categorized as either objective or subjective. Additionally, information on the use of CHM decoctions, CHM granules or CPMs in the RCTs was assessed and recorded.

### 2.5 RoB Assessment

We used the Cochrane RoB Tool to appraise the RoB in the included RCTs [24], including the following six domains independently: (i) random sequence generation, (ii) allocation concealment, (iii) participants and personnel blinding, (iv) outcome assessment blinding, (v) incomplete outcome data, and (vi) selective reporting. Each domain was classified as having a low, unclear, or high RoB. RoB is a key study‐level characteristic that can systematically affect reported outcomes. Therefore, instead of relying solely on RoB assessments from the original SRs, we independently evaluated the RoB of each RCT using a standardized approach. This allowed us to incorporate RoB into our analysis and consider it as a potential confounding factor when assessing the difference in outcomes across different orally administered CHM dosage forms [25].

### 2.6 Data Analysis

Data were presented as frequencies for categorical outcomes and as medians for continuous outcomes. Differences in treatment and adverse effect estimates between RCTs using different orally administered CHM dosage forms were estimated using a two‐step approach [16]. Meta‐epidemiological analyses were conducted to assess differences between the following pairwise comparisons: CHM decoctions vs. CHM granules, CHM decoctions vs. CPMs, and CPMs vs. CHM granules. Binary and continuous outcome data were analyzed respectively.

- Step 1: The effect estimates for CHM treatment effects and adverse effects were analyzed within each MA. The odds ratios (ORs) and standardized mean differences (SMDs) with 95% confidence intervals (CIs) were computed for binary and continuous outcomes, respectively. The OR >1 or SMD >0 represented more favorable treatment effects or more severe adverse effects of CHM compared to control arm. For each MA, random‐effect meta‐regression analyses were used to estimate the differences in treatment or adverse effects of CHM among RCTs with different orally administered CHM dosage forms [26]. The coefficients from each meta‐regression analysis indicated the impact of different orally administered CHM dosage forms on effect sizes, reported as ratios of odds ratios (RORs) for binary outcomes and differences in standardized mean differences (dSMDs) for continuous outcomes. The RORs compared OR from different group, and a consistent estimator that commonly applied in MAs [27]. Whereas dSMDs compared the SMDs among two studies or two groups and evaluated the if the effect size differ among two groups [28].
- Step 2: After extracting the coefficients from each MA, we further combined them using random‐effect models implemented via the meta package in R. Specifically, we used the DerSimonian and Laird method to estimate the between‐MA variance (τ^2^). Heterogeneity was assessed using the *I* ^2^ statistic τ^2^ [29, 30]. Levels of heterogeneity were classified as low (<25%), moderate (25%‐50%), and high (>50%) based on *I^2^* values [30]. A *p*‐value < 0.05 was considered statistically significant, except for heterogeneity test, where *p*‐value < 0.10 indicated significant heterogeneity [31].

### 2.7 Subgroup and Sensitivity Analysis

Subgroup analyses were conducted based on the study characteristics, such as clinical conditions, poutcome nature (objective vs. subjective), and funding support (with vs. without funding information). The RCT sample size, funding support, incorporation of SD, and the six RoB domains were adjusted for one by one in the meta‐regression model to control for potential confounders, due to the limited number of RCTs included in each MA. We further conducted a sensitivity analysis in which all potential confounders were controlled for simultaneously within a single model. All analyses were conducted using R version 4.2.2.

### 2.8 Publication Bias Assessment

A funnel plot was constructed to evaluate potential publication bias, and Egger's regression test was applied to assess symmetry for all outcomes that included more than ten studies [32].

## 3 RESULTS

### 3.1 Literature Search and Screening

**FIGURE 1:**
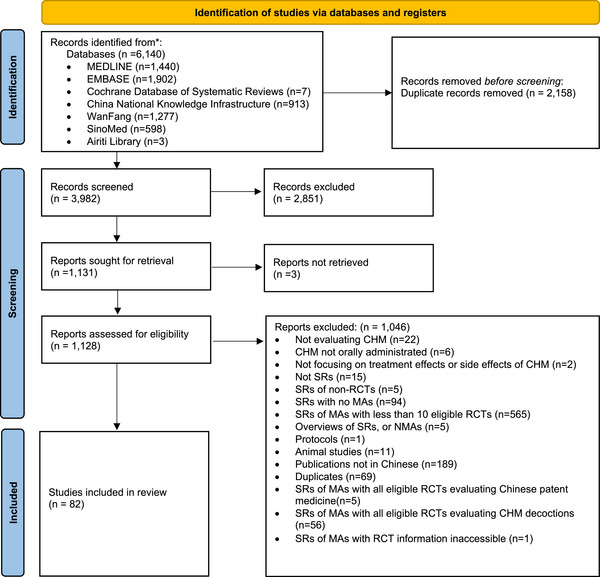
Flowchart of literature search and selection (from January 2021 to September 2022). CHM, Chinese herbal medicine; MAs, meta‐analysis; NMAs, network meta‐analyses; RCTs, randomized controlled trials; SRs, systematic reviews.

A total of 6140 studies were searched. Among them, 2158 records were identified as duplicates, and 2851 records were not included in this study based on title and abstract screening. Following a full‐text assessment and embedded RCT evaluation, 3 full texts were not retrieved and 1046 records were further excluded. Ultimately, 82 SRs fulfilled the eligibility criteria for inclusion in the study. In other words, 82 eligible MAs were included in this meta‐epidemiological study. The process of literature search and screening is illustrated in Figure 1, and a comprehensive list of the included SRs is available in Table S1.

### 3.2 Characteristics of Included Studies

The 82 MAs included in this study comprised 1263 eligible RCTs, involving a total of 111,179 participants (Tables 1 and 2). These MAs covered a diverse array of diseases, including diseases of the genitourinary system (20 SRs, 24.4%), circulatory system (18 SRs, 22.0%), digestive system (14 SRs, 17.1%), endocrine, nutritional, or metabolic disorders (6 SRs, 7.3%), along with various other conditions (24 SRs, 29.3%), as listed in Table 1. Among these 82 SRs of MAs, 44 (53.7%) were funded by non‐industry funding, while the remaining 38 (46.3%) did not providing information on funding sources.

**TABLE 1:** General characteristics of the 82 systematic reviews.

Characteristics	Results,^a^ *n* (%)
**Characteristics of the systematic reviews**	
Year of publication	
2021	57 (69.5)
2022	25 (30.5)
Clinical conditions classified by ICD‐11	
Diseases of the genitourinary system	20 (24.4)
Diseases of the circulatory system	18 (22.0)
Diseases of the digestive system	14 (17.1)
Endocrine, nutritional or metabolic diseases	6 (7.3)
Other conditions^b^	24 (29.3)
Funding support	
Non‐industry support	44 (53.7)
Not reported	38 (46.3)
Risk‐of‐bias assessment tool applied	
Cochrane Risk of Bias Tool	62 (75.6)
Both Cochrane Risk of Bias Tool and Jadad scale	12 (14.6)
Jadad scale	7 (8.5)
Cochrane Risk of Bias Tool 2.0	1 (1.2)
**Characteristics of meta‐analyses conducted in the systematic reviews**	
Median number of eligible RCTs per meta‐analysis, range	14, 10–34
No. of meta‐analyses reporting treatment effects	82 (100.0)
Treatment effect data	
Binary outcome	60 (73.2)
Continuous outcome	22 (26.8)
Treatment effect nature	
Subjective outcome	59 (72.0)
Objective outcome	23 (28.0)
No. of meta‐analyses reporting adverse effects	10 (12.2)
Adverse effect outcome data	
Binary outcome	10 (100.0)
Continuous outcome	0 (0.0)
Adverse effect outcome nature	
Subjective outcome	10 (100.0)
Objective outcome	0 (0.0)

Abbreviation: ICD‐11, International Classification of Diseases 11th Revision.

^a^ Values are frequency numbers (percentages) unless stated otherwise.

^b^ Other clinical conditions cover 11 categories, including sleep‐wake disorders (6, 7.3%), diseases of the immune system (4, 4.9%), mental, behavioral or neurodevelopmental disorders (3, 3.7%), diseases of the nervous system (3, 3.7%), diseases of the musculoskeletal system or connective tissue (3, 3.7%), neoplasms (1, 1.2%), diseases of the visual system (1, 1.2%), diseases of the respiratory system (1, 1.2%), symptoms, signs or clinical findings, not elsewhere classified (1, 1.2%), injury, poisoning or certain other consequences of external causes (1, 1.2%).

**TABLE 2:** General characteristics of the 1263 randomized controlled trials.^a^

Characteristics	Overall (*n* = 1263)	RCTs using CHM decoctions (*n* = 950)	RCTs using Chinese patent medicine (*n* = 186)	RCTs using CHM granules (*n* = 127)	*p* value
Median year of publication, range	2016, 1995–2021	2016, 1995–2021	2016, 2001–2021	2017, 2002–2021	0.026
Median follow‐up duration for the outcome of interest, range (weeks)	8, 0.7–60	8, 0.7‐52	8, 1–52	12, 2–60	<0.001
Total number of participants included in primary studies	111,179	81,937	18,050	11,192	NA
Median sample size, range	80, 30–430	80, 30–354	85, 30–357	66, 30–430	0.006
Publication language					
Chinese	1260 (99.8)	950 (100.0)	184 (98.9)	126 (99.2)	0.009
English	3 (0.2)	0 (0.0)	2 (1.1)	1 (0.8)	
Types of comparisons					
CHM + conventional treatment vs. conventional treatment	768 (60.8)	547 (57.6)	147 (79.0)	74 (58.3)	<0.001
CHM vs. conventional treatment	495 (39.2)	403 (42.4)	39 (21.0)	53 (41.7)	
Whether incorporating syndrome differentiation					
Yes	724 (57.3%)	567 (59.7%)	67 (36%)	90 (70.9%)	<0.001
No	539 (42.7%)	383 (40.3%)	119 (64%)	37 (29.1%)	
Types of RCTs					
Two‐arm	1208 (95.6)	921 (96.9)	167 (89.8)	120 (94.5)	<0.001
Multi‐arm	55 (4.4)	29 (3.1)	19 (10.2)	7 (5.5)	
Number of centers					
Single‐center	1170 (92.6)	882 (92.4)	174 (93.5)	118 (92.9)	0.051
Multi‐center	39 (3.1)	24 (2.5)	9 (4.8)	6 (4.7)	
Not reported	54 (4.3)	48 (5.1)	3 (1.6)	3 (2.4)	
Funding support					
Non‐industry support	291 (23.0)	209 (22.0)	43 (23.1)	39 (30.7)	0.032
Industry support	1 (0.1)	0 (0.0)	2 (1.1)	0 (0.0)	
Not reported	971 (76.9)	745 (78.1)	142 (76.3)	88(69.3)	

Abbreviations: CHM, Chinese herbal medicine; NA, not applicable; RCTs, randomized controlled trials.

^a^ Values are numbers (percentages) unless stated otherwise.

Regarding the RoB assessment of MAs, a substantial portion of the SRs (62, 75.6%) utilized the Cochrane RoB Tool, 12 SRs (14.6%) employed both the Cochrane RoB Tool and the Jadad scale, 7 SRs (8.5%) used the Jadad scale, and only one SR (1.2%) applied the Cochrane RoB Tool 2.0. The median number of RCTs contained in per MA was 14, with a range of 10 to 34. Among all the MAs included in this study, 60 (73.2%) MAs reported predominantly binary treatment‐effect outcomes, and 59 (72.0%) MAs reported subjective treatment‐effect outcome. Of the 60 (73.2%) MAs reporting binary outcomes, 55 (67.1%) reported the clinical efficacy rate, defined as the proportion of patients whose symptoms improved, resolved, or disappeared after treatment. As for the adverse effects related to CHM, only 10 (12.2%) MAs reported sufficient data for analysis, and all the effects were reported as binary and subjective.

The characteristics of the 1263 RCTs are summarized in Table 2. Among these, 950 (75.2%) used CHM decoctions, 186 (14.7%) used CPMs, and 127 (10.0%) used CHM granules. Among all the included RCTs, the publication years range from 1995 to 2021, with a median of 2016. The median of sample size among all included RCTs in this study was 80 participants, the RCTs with smallest sample size included only 30 participants, while the RCTs with the largest sample size reached 430 participants. Notably, RCTs using CHM granules had smaller sample sizes compared to those using the other two orally administered CHM dosage forms (*p* < 0.001). Nearly all RCTs (1260, 99.8%) were published in Chinese. 768 (60.8%) RCTs evaluated the comparison between CHM in conjunction with conventional treatment and conventional treatment, and 495 (39.2%) RCTs evaluated comparison between CHM and conventional treatment. More than half of the RCTs (724, 57.3%) incorporated syndrome differentiation. RCTs using CPMs had a much lower proportion of syndrome differentiation compared to those using CHM decoctions and CHM granules (36% for CPMs vs. 59.7% for CHM decoctions vs. 70.9% for CHM granules, *p* < 0.001). Most RCTs (1208, 95.6%) adopted a two‐arm design, and 1170 (92.6%) were single‐center studies. Additionally, 971 (76.9%) of the RCTs did not declare any funding sources.

### 3.3 RoB Assessment of Included RCTs

The included RCTs were found to have good performance regarding incomplete outcome data, with 90.6% having a low RoB. Specifically, RCTs using CHM decoctions had a higher proportion of the low RoB for incomplete outcome data (92.3%), compared to those using CPMs (87.6%) and using CHM granules (81.9%), with a statistically significant difference (*p* = 0.001).

However, the performances were unsatisfactory in the domains of allocation concealment and blinding of participants and personnel, with only 2.3% and 2.7% of the RCTs, were identified as low RoB. For random sequence generation and blinding of the outcome assessment, 47.7% and 31.2% of the RCTs, respectively, were rated as having a low RoB. Additionally, due to the lack of open‐access registration information or protocols, 96.1% of the RCTs were identified as unclear RoB for selective reporting. Details are presented in Table 3.

**TABLE 3:** Risk of Bias amongst 1263 randomized controlled trials.^a^

Risk of bias domains and risk levels	Overall (*n* = 1263)	RCTs using CHM decoctions (*n* = 950)	RCTs using Chinese patent medicine (*n* = 186)	RCTs using CHM granules (*n* = 127)	*p* value
Random sequence generation					
Low	603 (47.7%)	436 (45.9%)	83 (44.6%)	84 (66.1%)	<0.001
Unclear	605 (47.9%)	470 (49.5%)	95 (51.1%)	40 (31.5%)	
High	55 (4.4%)	44 (4.6%)	8 (4.3%)	3 (2.4%)	
Allocation concealment					
Low	29 (2.3%)	12 (1.3%)	7 (3.8%)	10 (7.9%)	<0.001
Unclear	1179 (93.3%)	894 (94.1%)	171 (91.9%)	114 (89.8%)	
High	55 (4.4%)	44 (4.6%)	8 (4.3%)	3 (2.4%)	
Blinding of participants and personnel					
Low	34 (2.7%)	17 (1.8%)	9 (4.8%)	8 (6.3%)	0.013
Unclear	1219 (96.5%)	925 (97.4%)	176 (94.6%)	118 (92.9%)	
High	10 (0.8%)	8 (0.8%)	1 (0.5%)	1 (0.8%)	
Blinding of outcome assessment					
Low	394 (31.2%)	268 (28.2%)	84 (45.2%)	42 (33.1%)	<0.001
Unclear	866 (68.6%)	679 (71.5%)	102 (54.8%)	85 (66.9%)	
High	3 (0.2%)	3 (0.3%)	0 (0%)	0 (0%)	
Incomplete outcome data					
Low	1144 (90.6%)	877 (92.3%)	163 (87.6%)	104 (81.9%)	0.001
Unclear	7 (0.6%)	3 (0.3%)	2 (1.1%)	2 (1.6%)	
High	112 (8.9%)	70 (7.4%)	21 (11.3%)	21 (16.5%)	
Selective outcome reporting					
Low	0 (0.0)	0 (0.0)	0 (0.0)	0 (0.0)	0.286
Unclear	1214 (96.1%)	917 (96.5%)	178 (95.7%)	119 (93.7%)	
High	49 (3.9%)	33 (3.5%)	8 (4.3%)	8 (6.3%)	

Abbreviations: CHM, Chinese herbal medicine; high, high risk of bias; low, low risk of bias; unclear, unclear risk of bias.

^a^ Values are numbers (percentages) unless stated otherwise.

### 3.4 Main Analysis, Subgroup Analysis, and Sensitivity Analysis

**FIGURE 2:**
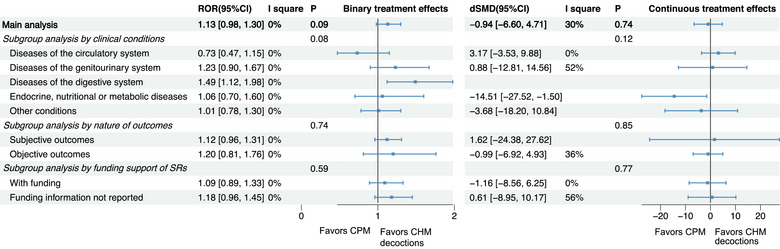
Main analysis and subgroup analyses of binary and continuous treatment effects of Chinese herbal medicine: Chinese patent medicine (ref) vs. CHM decoctions in RCTs. CHM, Chinese herbal medicine; CI, confidence interval; CPM, Chinese patent medicine; dSMD, difference in standardized mean difference; ROR, risk of odds ratio.

**FIGURE 3:**
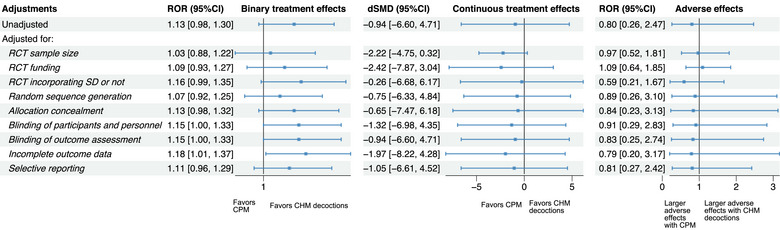
Sensitivity analyses of binary and continuous treatment effects, as well as adverse effects of Chinese herbal medicine: Chinese patent medicine (ref) vs. CHM decoctions in RCTs. CHM, Chinese herbal medicine; CI, confidence interval; CPM, Chinese patent medicine; dSMD, difference in standardized mean difference; RCT, randomized controlled trial; ROR, risk of odds ratio; SD, syndrome differentiation.

**FIGURE 4:**
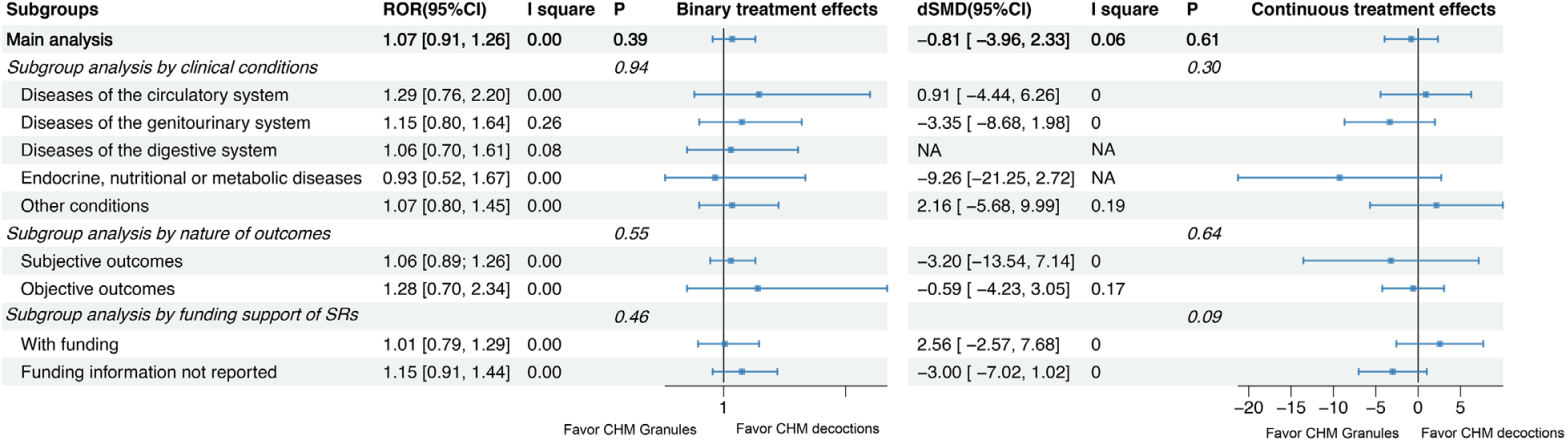
Main analysis and subgroup analyses of binary and continuous treatment effects of Chinese herbal medicine: CHM granules (ref) vs. CHM decoctions in RCTs. CHM, Chinese herbal medicine; CI, confidence interval; dSMD, difference in standardized mean difference; NA, not applicable; ROR, risk of odds ratio; SR, systematic review.

**FIGURE 5:**
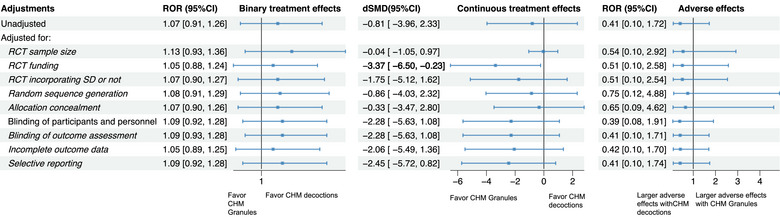
Sensitivity analyses of binary and continuous treatment effects, as well as adverse effects of Chinese herbal medicine: CHM granules (ref) vs. CHM decoctions in RCTs. CHM, Chinese herbal medicine; CI, confidence interval; dSMD, difference in standardized mean difference; RCT, randomized controlled trial; ROR, risk of odds ratio; SD, syndrome differentiation.

**FIGURE 6:**
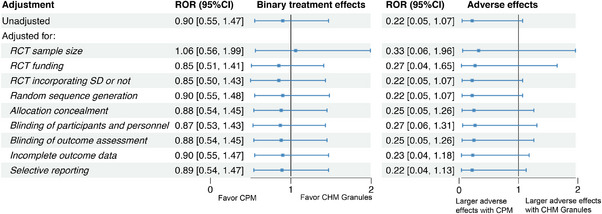
Sensitivity analyses of binary treatment effects and adverse effects of Chinese herbal medicine: Chinese patent medicine (ref) vs. CHM granules in RCTs. CHM, Chinese herbal medicine; CI, confidence interval; CPM, Chinese patent medicine; RCT, randomized controlled trial; ROR, risk of odds ratio; SD, syndrome differentiation.

Results of the meta‐epidemiological analyses assessing differences in effect estimates among three pairwise comparisons: CHM decoctions versus CHM granules, CHM decoctions versus CPMs, and CPMs versus CHM granules are presented in Figures 2, 3, 4, 5, 6 and Tables S2–S6.

#### 3.4.1 CHM Decoctions Versus CPMs

Figure 2 presents the results of the main and subgroup analyses comparing binary and continuous treatment effect estimates between RCTs using CHM decoctions and those using CPMs. Overall, no significant difference was observed in either binary treatment effects (ROR: 1.13, 95% CI: 0.98 to 1.30) or continuous treatment effects (dSMD: –0.94, 95% CI: –6.60 to 4.71). Subgroup analysis further revealed that RCTs on digestive diseases tended to report larger binary treatment effect estimates when using CHM decoctions than using CPMs (ROR: 1.49, 95% CI: 1.12 to 1.98). Conversely, RCTs on endocrine, nutritional or metabolic diseases tended to report larger continuous treatment effects when using CPMs than using CHM decoctions (dSMD: –14.51, 95% CI: –27.52 to –1.50) (Figure 2).

Sensitivity analysis revealed that after adjusting for incomplete outcome data, decoctions were associated with slightly larger binary treatment effect estimates compared to CPMs (ROR: 1.18, 95% CI: 1.01 to 1.37) (Figure 3, Table S3). For continuous treatment effects, when adjusting for all potential confounders, CPMs were associated with greater continuous treatment effects than CHM decoctions (dSMD: –3.72, 95% CI: –6.91 to –0.52, *I* ^2^: 65%) (Table S3).

No significant differences were observed for adverse effects (ROR: 0.80, 95% CI: 0.26 to 2.47) (Figure 3).

#### 3.4.2 CHM Decoctions Versus CHM Granules

Regarding the comparison between CHM decoctions and CHM granules, no significant differences were observed in either binary or continuous treatment effects as well (Figure 4). Nevertheless, sensitivity analysis showed that, after adjusting for funding, CHM granules were associated with larger continuous treatment effects compared to CHM decoctions (dSMD: –3.37, 95% CI: –6.50 to –0.23) (Figure 5).

Regarding adverse effects, the results showed no significant differences (ROR: 0.41, 95% CI: 0.10 to 1.72) between RCTs using CHM decoctions and those using CHM granules (Figure 5).

#### 3.4.3 CPMs Versus CHM Granules

For CPMs versus CHM granules, only binary treatment effects and adverse effects were analyzed due to limited data availability. The results indicated no significant difference in binary treatment effects (ROR: 0.90, 95% CI: 0.55 to 1.47) or adverse effects (ROR: 0.22, 95% CI: 0.05 to 1.07) (Figure 6). Sensitivity analysis further confirmed the robustness of these findings. Subgroup analyses were not conducted due to insufficient data.

Regarding adverse effects, the results showed no significant differences (ROR: 0.22, 95% CI: 0.05 to 1.07) between any of the pairwise comparisons (Figure 6).

### 3.5 Publication Bias

The results of the publication bias analysis are provided in Table S7 and Supplementary Material C. Visual inspection of the funnel plot showed no signs of asymmetry, and Egger's test indicated no significant publication bias for all outcomes (*p* > 0.05).

## 4 Discussion

This meta‐epidemiological study analyzed 82 SRs to evaluate whether different orally administered CHM dosage forms, specifically, CHM decoctions, CPMs, and CHM granules, are associated with variations in treatment effects and adverse effects in CHM RCTs. Overall, differences in binary treatment outcomes, continuous treatment effects, and adverse effects among these orally administered CHM dosage forms were minimal and statistically insignificant. Sensitivity and subgroup analyses indicated some variations: CHM decoctions tend to be associated with slightly larger binary treatment effect than CPMs, particularly in RCTs focused on digestive diseases, while CPMs tend to show larger continuous treatment effects in trials on endocrine and metabolic diseases than CHM decoctions. However, when adjusting for confounders, CPMs were associated with greater continuous treatment effects, and CHM granules showed larger estimates than decoctions after adjusting for funding support.

These results are consistent with previous studies reporting minimal differences in treatment effect estimates among various orally administered CHM dosage forms. Earlier SRs similarly found no significant difference in treatment effectiveness and safety between granules and decoctions [2, 4]. A prior meta‐epidemiological study reported that CHM decoctions might be associated with slightly larger binary treatment effects than CPMs, particularly in trials without funding support [33]. The comparable treatment effects observed across different orally administered CHM dosage forms may reflect similar bioavailability and active ingredient composition when derived from the same herbal materials. For instance, an analysis of the chemical consistency and anti‐tumor activity of Huangqi‐Ezhu concentrated granules and decoction found that the level and types of active components in both forms were essentially the same [34]. However, a fingerprint comparison of Gegen Qinlian preparations in three orally administered CHM dosage forms—decoction, dispensing granule, and pill—revealed that differences in orally administered CHM dosage forms may lead to variations in the content of active ingredients, even when using the same batches of raw materials [35]. The complexity of Chinese herbal formulas, with various chemical reactions during preparation, and differences in manufacturing processes, such as boiling, drying and granulation, may impact dissolution rates and alter the proportions of available compounds [2]. Previous studies have shown that many phytochemical components undergo physical and chemical transformations during decocting, which may influence clinical effectiveness [36, 37]. Given these considerations, future research should evaluate these chemical reactions during preparation and assess the consistency of chemical composition across different dosage forms to further validate our findings.

Specific differences noted in the subgroup analyses, such as larger effect estimates for decoctions in digestive disease trials and CPMs in endocrine disorder trials, may relate to differences in absorption and delivery mechanisms. Decoctions, being liquid‐based might facilitate faster absorption and action [38], which is particularly beneficial in digestive conditions. In contrast, CPMs that are in pill or capsule form requires dissolution of the active compounds in the gut, potentially offering a slower and more sustained release of active compounds, which might be advantageous in managing endocrine and metabolic diseases. This interpretation aligns with recent findings on the interaction between diabetes mellitus and gut microbiota, where dietary components and CPM active compounds influence gut microflora composition and metabolic outcomes [39]. Moreover, CPMs packaged in a modified release form may also support the proliferation of beneficial gut bacteria and the production of metabolites such as short‐chain fatty acids, which are known to improve insulin resistance and glycemic control [40]. However, it is important to note that the included SRs in our study provided limited information on the release profiles of the dosage forms. Some CPMs in pill or capsule form may indeed offer an immediate release of active compounds. Given the lack of direct comparative studies between sustained‐release CPMs and decoctions in terms of their effects on gut microbiome modulation, further research is needed to explore the mechanisms of subgroup difference in greater detail.

Sensitivity analyses supported most findings, with two notable exceptions. After adjusting for all confounders, CPMs were associated with relatively larger continuous treatment effects, though high heterogeneity was observed, indicating the need for cautious interpretation. CHM granules were also found to be associated with larger treatment effects than decoctions when adjusting for funding. These trends may be attributed to the standardized production processes and consistent dosing of CPMs and granules [41]. In particular, quality control is a critical aspect of CPM manufacturing [42], which may contribute to enhanced treatment reproducibility and better patient adherence. However, further studies are needed to confirm these observations and explore underlying mechanisms.

For orally administered drugs, pharmacological action relies on adequate intestinal absorption and distribution before elimination [43]. Future research should assess intestinal absorption assays and bioavailability across different orally administered CHM dosage forms using a range of in‐vivo, in‐vitro, in‐situ and in‐silico models [43]. Longitudinal studies examining the long‐term treatment effects and safety across diverse populations and health conditions could provide deeper insights into their therapeutic potential and limitations. In this study, though the absence of significant differences in adverse effects across different orally administered CHM dosage forms, more thorough reporting and investigation are necessary to confirm these findings.

The interaction between CHM and the gut microbiota has been proposed as a possible key mechanism underlying the therapeutic effects of CHM [44]. Studies have shown that TCM plays a vital role in promoting health and treating disease by interacting with the gut microbiota [45]. CHMs may influence the host gut environment in various ways, including inhibiting pathogenic bacteria, promoting the growth of probiotics, regulating key microbial metabolites, and maintaining the integrity of the intestinal barrier [46, 47, 48]. While this mechanism is not the primary focus of our study, we briefly included it to provide context for the observed treatment effects. Future research should further explore the possible differences in therapeutic effects of CHM with different dosage forms and their interaction with gut microbiota.

Improving study reporting is crucial for advancing research in this field. The CONSORT (Consolidated Standards of Reporting Trials) Extension for CHM Formulas 2017 recommends that trialists provide detailed reports of interventions for each group, including how and when they were administered, to allow replication [49]. While this guideline emphasizes the need to report details regarding the composition, dosage, effectiveness, safety, and quality control of formulas in CPMs, it does not specifically mandate the reporting of dosage form characteristics, such as whether the studied pills or capsules are immediate‐release or sustained‐release. Therefore, in addition to adhering to the CONSORT guidelines, future trialists and reviewers should emphasize including such information to facilitate pharmacodynamic studies that compare the effects of different dosage forms with varying release rates.

Given the minimal and statistically insignificant differences observed among CHM decoctions, CPMs, and CHM granules in terms of treatment and adverse effects, except for specific indications such as digestive and endocrine disorders, TCM practitioners may base their prescription decisions on factors such as cost, availability, and patient preference. For example, a previous study found that the retail price of single‐herb concentrated granules is approximately 30% higher than traditional decoctions, primarily due to the inclusion of decoction preparation costs, yet it remains significantly lower than the cost of equivalent doses in CPMs [50]. Therefore, considerations such as cost‐effectiveness may guide decisions. Additionally, the feasibility of different dosage forms among specific patient populations should be considered. For example, patients with busy schedules may find it inconvenient to prepare decoctions, making more user‐friendly dosage forms like CPMs or granules a preferable option [51].

While this meta‐epidemiological study provides a broad overview of how different CHM dosage forms are associated with reported treatment effects and adverse events across diverse conditions, further research is warranted to validate these findings in more controlled settings. Specifically, future studies could consider conducting head‐to‐head RCTs that compare decoctions, granules, and CPMs using identical herbal formulas and targeting patients with the same TCM syndrome diagnosis. Additionally, network MAs focusing on specific diseases or syndromes may offer more granular insights into the comparative effectiveness and safety of different dosage forms. These approaches would complement our findings and help establish more robust evidence for clinical decision‐making in CHM practice.

This study provided valuable insights into the association between dosage forms and the treatment and adverse effect estimates in CHM RCTs. The inclusion of a large number of recently published SRs and MAs allowed for a comprehensive analysis across a broad range of clinical conditions, offering a representative sample for addressing the research question. The study's rigor was further enhanced by stringent inclusion criteria during the selection process, ensuring clear comparisons between trials involving different CHM dosage forms. Additionally, the use of a standardized data extraction form and audit procedure minimized errors, while sensitivity and subgroup analyses confirmed the robustness of findings.

However, the study has several limitations. Despite conducting sensitivity analyses that considered potential confounders such as sample size, funding support, and RoB in RCTs were conducted, unobserved confounding factors (such as the number of centers involved in the trials) may still exist and were not accounted for. The reliance on published data limits the ability to consider unpublished studies or those not registered in accessible databases, potentially introducing publication bias. The assessment of RoB highlighted areas for improvement in the design and reporting of CHM RCTs, especially in the area of random sequence generation, allocation concealment, participants and personnel blinding, and outcome assessment blinding. These methodological shortcomings could contribute to the overestimation of treatment effects and limit the generalizability of findings [52]. Moreover, certain orally administered CHM dosage forms may be preferentially prescribed to specific patient groups with distinct clinical characteristics. As our inclusion criteria required SRs to contain MAs comparing at least two orally administered CHM dosage forms, reviews focusing exclusively on a single orally administered dosage form were excluded. This may limit the clinical representativeness of our findings, and caution is warranted when interpreting the results in disease‐specific or patient‐specific contexts. Addressing gaps in data and improving the design and reporting of CHM studies will enhance the reliability and applicability of future research findings.

This meta‐epidemiological study found that CHM decoctions, CPMs, and granules are associated with minimal differences in reported treatment and adverse effect estimates in CHM RCTs. However, CHM decoctions tend to show slightly larger binary treatment outcomes compared to CPMs, particularly in RCTs on digestive diseases, while CPMs are associated with larger continuous treatment effects in trials on endocrine and metabolic diseases. When adjusting for confounders, CPMs demonstrate relatively greater continuous treatment effect estimates, and CHM granules are associated with larger estimates than decoctions when adjusted for funding. These findings contribute to the ongoing discussion on the relative merits of different orally administered CHM dosage forms and highlight the need for further research to confirm these results and clarify the mechanisms underlying their therapeutic effects and safety.

## Funding

This work was supported by the Chinese Medicine Development Fund of the Hong Kong SAR (*No*. 21B2/018A). The funders had no role in the study design or collection, analysis, interpretation of data, writing of the report, or decision to submit the article for publication.

## Conflicts of Interest

The authors declared no potential conflicts of interest with respect to the research, authorship, and/or publication of this article.

## Supporting information




**Supplementary Material A**: Search strategies and results for systematic reviews of Chinese herbal medicine.
**Supplementary Material B**: Pre‐specified data extraction form.
**Supplementary Material C**: Funnel plots for all outcomes with over 10 studies.
**Table S1**: List of included 82 systematic reviews.
**Table S2**: Details of main analysis and subgroup analysis of binary and continuous treatment effects of Chinese herbal medicine: Chinese patent medicine (ref) vs. CHM decoctions in RCTs.
**Table S3**: Details of sensitivity analysis of binary and continuous treatment effects, as well as adverse effects of Chinese herbal medicine: Chinese patent medicine (ref) vs. CHM decoctions in RCTs.
**Table S4**: Details of main analysis and subgroup analysis of binary and continuous treatment effects of Chinese herbal medicine: CHM granules (ref) vs. CHM decoctions in RCTs.
**Table S5**: Details of sensitivity analysis of binary and continuous treatment effects, as well as adverse effects of Chinese herbal medicine: CHM granules (ref) vs. CHM decoctions in RCTs.
**Table S6**: Details of sensitivity analysis of binary and adverse effects of Chinese herbal medicine: Chinese patent medicine (ref) vs. CHM granules in RCTs.
**Table S7**: Results of Egger's test for all outcomes with over 10 studies.
**Figure S1**: Funnel plot for main analysis of binary treatment effects: CHM granules vs. CHM decoction. CHM, Chinese herbal medicine.
**Figure S2**: Funnel plot for sensitivity analysis of binary treatment effects adjusted for all potential covariates: CHM granules vs. CHM decoction. CHM, Chinese herbal medicine.
**Figure S3**: Funnel plot for main analysis of continuous treatment effects: CHM granules vs. CHM decoction. CHM, Chinese herbal medicine.
**Figure S4**: Funnel plot for sensitivity analysis of continuous treatment effects adjusted for all potential covariates: CHM granules vs. CHM decoction. CHM, Chinese herbal medicine.
**Figure S5**: Funnel plot for main analysis of binary treatment effects: Chinese patent medicine vs. CHM decoction. CHM, Chinese herbal medicine.
**Figure S6**: Funnel plot for sensitivity analysis of binary treatment effects adjusted for all potential covariates: Chinese patent medicine vs. CHM decoction. CHM, Chinese herbal medicine.
**Figure S7**: Funnel plot for main analysis of continuous treatment effects: Chinese patent medicine vs. CHM decoction. CHM, Chinese herbal medicine.
**Figure S8**: Funnel plot for sensitivity analysis of continuous treatment effects adjusted for all potential covariates: Chinese patent medicine vs. CHM decoction. CHM, Chinese herbal medicine.

## Data Availability

The data that support the findings of this study are available on request from the corresponding authors.
