# Supplementary material for: Impact of Different Dosage Forms of Orally Administered Chinese Herbal Medicine on Treatment and Adverse Effect Estimates in Randomized Controlled Trials: A Meta‐Epidemiological Study
Source: J Evid Based Med. 2026 Jan 31;19(1):e70115. doi: 10.1111/jebm.70115 (PMC13039769; doi:10.1111/jebm.70115)
Supplement: Supplementary file 1 — Supplementary Material A: Search strategies and results for systematic reviews of Chinese herbal medicine. Supplementary Material B: Pre‐specified data extraction form. Supplementary Material C: Funnel plots for all outcomes with over 10 studies. Table S1: List of included 82 systematic reviews. Table S2: Details of main analysis and subgroup analysis of binary and continuous treatment effects of Chinese herbal medicine: Chinese patent medicine (ref) vs. CHM decoctions in RCTs. Table S3: Details of sensitivity analysis of binary and continuous treatment effects, as well as adverse effects of Chinese herbal medicine: Chinese patent medicine (ref) vs. CHM decoctions in RCTs. Table S4: Details of main analysis and subgroup analysis of binary and continuous treatment effects of Chinese herbal medicine: CHM granules (ref) vs. CHM decoctions in RCTs. Table S5: Details of sensitivity analysis of binary and continuous treatment effects, as well as adverse effects of Chinese herbal medicine: CHM granules (ref) vs. CHM decoctions in RCTs. Table S6: Details of sensitivity analysis of binary and adverse effects of Chinese herbal medicine: Chinese patent medicine (ref) vs. CHM granules in RCTs. Table S7: Results of Egger's test for all outcomes with over 10 studies. Figure S1: Funnel plot for main analysis of binary treatment effects: CHM granules vs. CHM decoction. CHM, Chinese herbal medicine. Figure S2: Funnel plot for sensitivity analysis of binary treatment effects adjusted for all potential covariates: CHM granules vs. CHM decoction. CHM, Chinese herbal medicine. Figure S3: Funnel plot for main analysis of continuous treatment effects: CHM granules vs. CHM decoction. CHM, Chinese herbal medicine. Figure S4: Funnel plot for sensitivity analysis of continuous treatment effects adjusted for all potential covariates: CHM granules vs. CHM decoction. CHM, Chinese herbal medicine. Figure S5: Funnel plot for main analysis of binary treatment effects: Chinese patent medicine vs. CH [file JEBM-19-0-s001.docx]

**Supplementary Materials**

Supplementary Material A. Search strategies and results for systematic reviews of Chinese herbal medicine

1. MEDLINE from 2021 to September 15, 2023

| **#** | **Search Statement** | **Results** |
| --- | --- | --- |
| 1 | MEDLINE.tw. | 151,670 |
| 2 | systematic review.tw. | 234,158 |
| 3 | meta analysis.pt. | 166,359 |
| 4 | 1 or 2 or 3 | 384,169 |
| 5 | exp Drugs, Chinese Herbal/ | 50,470 |
| 6 | Chinese herb*.mp. | 57,141 |
| 7 | exp Medicine, Chinese Traditional/ | 22,719 |
| 8 | Traditional Chinese medic*.mp. | 31,558 |
| 9 | exp Phytotherapy/ | 41,753 |
| 10 | phytother*.mp. | 42,577 |
| 11 | (chinese adj5 (traditional or medic*)).mp. | 64,919 |
| 12 | (herbs or herbal).mp. | 96,798 |
| 13 | (plant or plants).mp. | 964,037 |
| 14 | (traditional adj5 medic*).mp. | 87,222 |
| 15 | 5 or 6 or 7 or 8 or 9 or 10 or 11 or 12 or 13 or 14 | 1,083,477 |
| 16 | 4 and 15 | 9,091 |
| 17 | limit 16 to (humans and yr="2021 -Current") | 1,440 |

1. Embase from 2021 to September 15, 2023

| **#** | **Search Statement** | **Results** |
| --- | --- | --- |
| 1 | meta-analysis.tw. | 268,126 |
| 2 | systematic review.tw. | 285,343 |
| 3 | 1 or 2 | 427,432 |
| 4 | exp Chinese medicine/ | 64,102 |
| 5 | exp oriental medicine/ | 2,729 |
| 6 | exp herbaceous agent/ | 58,166 |
| 7 | exp medicinal plant/ | 276,697 |
| 8 | exp Chinese herb/ | 5,151 |
| 9 | Chinese medic*.mp. | 82,420 |
| 10 | oriental medic*.mp. | 4,211 |
| 11 | herbaceous agent.mp. | 58,168 |
| 12 | medicinal plant*.mp. | 107,299 |
| 13 | Chinese herb*.mp. | 19,646 |
| 14 | herb*.mp. | 218,986 |
| 15 | 4 or 5 or 6 or 7 or 8 or 9 or 10 or 11 or 12 or 13 or 14 | 498,461 |
| 16 | 3 and 15 | 7,557 |
| 17 | limit 16 to (human and yr="2021 -Current") | 1,902 |

1. Cochrane Database of Systematic Reviews from 2021 to September 15, 2023

“Chinese herb*” or “herb*” or “Traditional Chinese medic*” or “phytother*” or “(chinese adj5 (traditional or medic*))” or “(plant or plants)” or “(traditional adj5 medic*)” or “Chinese medic*” or “oriental medic*” or “herbaceous agent” or “medicinal plant*” with Cochrane Library publication date from Jan 2021 to Sep 2022, in Cochrane Reviews yielded 7 citations.

Search syntax and English translation in the databases in Chinese

| **Original text** | **English version** |
| --- | --- |
| **(iv) China National Knowledge Infrastructure (CNKI) from 2021 to September 15, 2023**  (((((((((((题名= '中医') OR (题名= '中医学')) OR (题名= '中药')) OR (题名= '草药')) OR (题名= '中草药')) OR (题名= '中成药')) OR (题名= '中医药')) OR (题名= '方剂')) OR (题名= '中西医')) OR (题名= '汤')) AND ((((((((题名= '系统评价') OR (题名= '系统综述')) OR (题名= '荟萃分析')) OR (题名= 'Meta分析')) OR (题名= '元分析')) OR (题名= '循证研究')) OR (题名= '集合分析')) OR (题名= '综合分析'))) limit time from Jan 2021 to Sep 15 2022, yielded 913 citations. | **(iv) China National Knowledge Infrastructure (CNKI) from 2021 to September 15, 2023**  (((((((((((Title='Chinese Medicine') OR (Title='Traditional Chinese Medicine')) OR (Title='Herbal Medicine')) OR (Title= ‘Herbal Materia’)) OR (Title=Pattern Medicine')) OR (Title='Chinese Pattern Medicine')) OR (Title='Proprietary Chinese Medicine')) OR (Title='Prescription')) OR (Title='Decoction)) AND ((((((((Title='Systematic Review') OR (Title='Literature Review')) OR (Title = 'Meta-analysis')) OR (Title = 'Meta-synthesis')) OR (Title = 'Evidence-based Research')) OR (Title = 'Ensemble Analysis')) OR (Title = 'Synthesis Analysis'))) limit time from Jan 2021 to Sep 15 2022, yielded 913 citations. |
| (v) WanFang from 2021 to September 15, 2023  (题名:(系统评价 or 系统综述 or 荟萃分析 or Meta分析 or 元分析 or 循证研究 or 集合分析 or 综合分析) and 题名:(中医 or 中医学 or 中药 or 草药 or 中草药 or 中成药 or 中医药 or 方剂 or 中西医 or 汤)) limit time from Jan 2021 to Sep 15 2022, yielded 1,277 citations. | (v) WanFang from 2021 to September 15, 2023  (Title: (Systematic Review or Literature Review or Meta-analysis or Meta-synthesis or Evidence-based Research or Ensemble Analysis or Synthesis) and Title: (Traditional Chinese Medicine or Chinese Medicine or Herbal Medicine or Herbal Materia Medica or Pattern Medicine or Proprietary Chinese Medicine or Prescription or Decoction)) limit time from Jan 2021 to Sep 15 2022, yielded 1,277 citations. |
| (vi) Chinese Biomedical Literature Database from 2021 to September 15, 2023  ("系统评价" [中文标题]) OR ("系统综述" [中文标题]) OR ("荟萃分析" [中文标题]) OR ("Meta分析" [中文标题]) OR ("元分析" [中文标题]) OR ("循证研究" [中文标题]) OR ("集合分析" [中文标题]) OR ("综合分析" [中文标题]) AND ("中医" [中文标题]) OR ("中医学" [中文标题]) OR ("中药" [中文标题]) OR ("草药" [中文标题]) OR ("中草药" [中文标题]) OR ("中成药" [中文标题]) OR ("中医药" [中文标题]) OR ("方剂" [中文标题]) OR ("中西药" [中文标题]) OR ("汤" [中文标题]) limit time from Jan 2021 to Sep 15 2022, yielded 598 citations. | (vi) Chinese Biomedical Literature Database from 2021 to September 15, 2023  ("Systematic review" [Chinese title]) OR ("Literature review" [Chinese title]) OR ("meta-analysis" [Chinese title]) OR ("Evidence-based Research" [Chinese Title]) OR ("Ensemble Analysis" [Chinese Title]) OR ("Comprehensive Analysis" [Chinese Title]) AND ("Chinese Medicine" [Chinese Title]) OR ("Traditional Chinese Medicine" [Chinese Title]) OR ("Herbal Medicine" [Chinese title]) OR ("Herbal Materia Medica" [Chinese title]) OR ("Chinese Pattern Medicine" [Chinese title]) OR ("Prescription" [Chinese title]) OR ("decoction" [Chinese title]). limit time from Jan 2021 to Sep 15 2022, yielded 598 citations. |
| (vii) Airiti Library from 2021 to September 15, 2023  ((((((( [DN]:(系统评价) OR [DN]:(系统综述)) OR [DN]:(荟萃分析)) OR [DN]:(Meta)) OR [DN]:(实证)) OR [DN]:(循证研究)) OR [DN]:(整合分析)) OR [DN]:(系统性文献回顾)) AND (((((((((( [DN]:(中医) OR [DN]:(中医学)) OR [DN]:(中药)) OR [DN]:(草药)) OR [DN]:(中草药)) OR [DN]:(中成药)) OR [DN]:(中医药)) OR [DN]:(方剂)) OR [DN]:(中西医)) OR [DN]:(汤)) OR ((((((((( [DN]:(丸) OR [DN]:(散)) OR [DN]:(膏)) OR [DN]:(成药)) OR [DN]:(本草)) OR [DN]:(成药)) OR [DN]:(草本药)) OR [DN]:(植物药)) OR [DN]:(饮片)) OR [DN]:(民间方))) limit time from Jan 2021 to Sep 15 2022, yielded 3 citations. | (vii) Airiti Library from 2021 to September 15, 2023  (((((([DN]:(Systematic Review) OR [DN]:(Literature review)) OR [DN]:(Meta-analysis)) OR [DN]:(Meta)) OR [DN]:(Empirical)) OR [DN]:(Evidence-Based Research)) OR [DN]:(Meso-analysis)) OR [DN]:(Systematic Literature Review)) AND ((((((((([DN]:( Chinese Medicine) OR [DN]:(Traditional Chinese Medicine)) OR [DN]:(Traditional Chinese Herbal Medicine)) OR [DN]:(Traditional Chinese Pattern Medicine)) OR [DN]:(Formulas)) OR [DN]:(Decoctions)) OR ((((((((([DN]:(Pills) OR [DN]:(Powders)) OR [DN]:(Ointments)) OR [DN]:(Pattern Medicines)) OR [DN]:( Herbal Materia Medica) OR [DN]:( Botanical Medicine) OR [DN]:( Herbal Pieces) OR [DN]:(Plant Medicine) OR [DN]:(Decoction Pieces) OR [DN]:(Folk Prescriptions) limit time from Jan 2021 to Sep 15 2022, yielded 3 citations. |

Supplementary Material B. Pre-specified data extraction form

**Part 1. Characteristics of systematic reviews**

(1) Year of publication: ______

(2) Clinical condition: ______

(3) Classification of disease: ______ (based on International Classification of Diseases, ICD-11)

1. Certain infectious or parasitic diseases;
2. Neoplasms;
3. Diseases of the blood or blood-forming organs;
4. Diseases of the immune system;
5. Endocrine, nutritional or metabolic diseases;
6. Mental, behavioral or neurodevelopmental disorders;
7. Sleep-wake disorders;
8. Diseases of the nervous system;
9. Diseases of the visual system;
10. Diseases of the ear or mastoid process;
11. Diseases of the circulatory system;
12. Diseases of the respiratory system;
13. Diseases of the digestive system;
14. Diseases of the skin;
15. Diseases of the musculoskeletal system or connective tissue;
16. Diseases of the genitourinary system;
17. Conditions related to sexual health;
18. Pregnancy, childbirth or the puerperium;
19. Certain conditions originating in the perinatal period;
20. Developmental anomalies;
21. Symptoms, signs or clinical findings, not elsewhere classified;
22. Injury, poisoning or certain other consequences of external causes;
23. External causes of morbidity or mortality;
24. Factors influencing health status or contact with health services.

(4) Funding support: ______

1. Industry support (Funding from any enterprises, commercial companies);
2. Non-industry support (Funding from governmental organization or non-governmental organization, such as charity institution, social association and individual donation);
3. Both industry and non-industry support (Funding from both sources 1 and 2);
4. No funding support (Authors reported no funding support);
5. Not reported (Absence of funding support information in the manuscript).

(5) Risk-of-bias assessment tool of included studies: ______

1. Cochrane risk-of-bias tool;
2. Cochrane risk-of-bias tool 2.0;
3. Jadad scale;
4. Pedro Scale;
5. Delphi list;
6. Others;
7. More than one tool;
8. Not report name of tool;
9. No quality assessment.

**Part 2. Characteristics of meta-analysis**

1. Number of eligible RCTs included in each meta-analysis: ______
2. Treatment effect outcomes reported or not: ______ 1) Yes; 2) No
3. Adverse effect outcomes reported or not: ______ 1) Yes; 2) No

**Part 3. Characteristics of randomized controlled trial**

1. Types of comparison: ______
   1. CHM vs conventional treatment;
   2. CHM ＋conventional treatment vs conventional treatment;
   3. CHM vs Placebo.
2. Types of Chinese herbal medicine:
3. Prescribed Chinese materia medica;
4. Chinese patent medicine;
5. Prescribed CHM Granules;
6. Whether RCTs incorporated syndrome differentiation: ______

1) Yes; 2) No

1. Publication language: ______

1) English; 2) Chinese

1. Funding support: ______
2. Industry support (Funding from any enterprises, commercial companies);
3. Non-industry support (Funding from governmental organization or non-governmental organization, such as charity institution, professional societies or individual donation);
4. Both industry and non-industry support (Funding from both sources 1 and 2);
5. No funding support (Authors reported no funding support);
6. Not reported (Absence of funding support information in the manuscript).
7. Center status: ______
   1. Single-center;
   2. Multi-center;
   3. Not reported.
8. Number of arms: ______
9. Two-arm;
10. Multiple-arm
11. Overall sample size: ______
12. Nature of treatment effect outcomes: ______
13. Objective outcomes (Outcomes not influenced by personal perspectives or preferences. e.g., All-cause mortality, childbirth rate, outcomes measured based on a recognized laboratory procedure or electronic equipment);
14. Subjective outcomes (Outcomes based on or influenced by personal perspectives or preferences, physician-assessed disease outcomes or outcomes defined and assessed by researchers).
15. Treatment outcome type: ______
16. Binary treatment effect outcome:

Number of events in treatment group: ______

Total number of patients in treatment group: ______

Number of events in control group: ______

Total number of patients in control group: ______

1. Continuous treatment effect outcome:

Mean and standard deviation in treatment group: ______

Total number of patients in treatment group: ______

Mean and standard deviation in control group: ______

Total number of patients in control group: ______

1. Nature of adverse effect outcomes: ______
2. Objective outcomes (Outcomes not influenced by personal perspectives or preferences. e.g., All-cause mortality, childbirth rate, outcomes measured based on a recognized laboratory procedure or electronic equipment, etc.);
3. Subjective outcomes (Outcomes based on or influenced by personal perspectives or preferences, physician-assessed disease outcomes or outcomes defined and assessed by researchers).
4. Adverse effect outcome type: ______
5. Binary adverse effect outcome:

Number of events in treatment group: ______

Total number of patients in treatment group: ______

Number of events in control group: ______

Total number of patients in control group: ______

1. Continuous adverse effect outcome:

Mean and standard deviation in treatment group: ______

Total number of patients in treatment group: ______

Mean and standard deviation in control group: ______

Total number of patients in control group: ______

Supplementary Table 1. List of included 82 systematic reviews

| **SRID** | **Authors** | **Year** | **SR title** | **Journal of publication** |
| --- | --- | --- | --- | --- |
| 1 | 尹爱武;万新;李秋 | 2022 | 利湿活血中药治疗慢性前列腺炎的Meta分析 | 中医临床研究 |
| 2 | 于林童;陶诗怡;逯金金;万洁;冯汝丽;曲文白;林谦;史大卓 | 2021 | 中药治疗微血管性心绞痛的Meta分析 | 中国中西医结合急救杂志 |
| 3 | 伍早霞 | 2021 | 黄连温胆汤治疗失眠临床疗效的Meta分析 | 陕西中医药大学（硕士学位论文） |
| 4 | 冀虹晓 | 2021 | 基于Meta分析探讨中药治疗围绝经期功血的有效性及安全性 | 黑龙江中医药大学（硕士学位论文） |
| 5 | 冯汝丽;崔晓云;邓宇童;曲信彦;毛天诗;谢龙;李星;刘静;高群;林谦 | 2021 | 口服中药治疗不稳定型心绞痛患者远期预后及疗效的Meta分析 | 世界中医药 |
| 6 | 冯汝丽;曲文白;曲信彦;于林童;毛天诗;万洁;逯金金;李岩;贾文浩;林谦 | 2021 | 口服中药治疗稳定型心绞痛疗效及安全性的Meta分析 | 世界中医药 |
| 7 | 刘凡;李双翼;郭洁;时昭红 | 2022 | 中药复方治疗非糜烂性胃食管反流病的随机对照试验系统评价与Meta分析 | 世界中医药 |
| 8 | 刘恭雪;曹焕泽;蔡平平 | 2021 | 中药复方治疗卵巢储备功能减退Meta分析 | 河南中医 |
| 9 | 刘雪婷 | 2021 | 中药治疗抽动障碍的Meta分析及袁梦石教授经验总结 | 湖南中医药大学（硕士学位论文） |
| 10 | 卫宇帆;张运克;樊飞燕;李双利;古春青 | 2022 | 中药复方治疗帕金森病睡眠障碍的Meta分析及基础用药数据挖掘 | 中医学报 |
| 11 | 叶茜 | 2021 | 中药复方治疗特发性膜性肾病的Meta分析及网络药理学研究 | 南京中医药大学（硕士学位论文） |
| 12 | 吕咪;刘平;张坤漓;史中斐;郑艺君;王凤云 | 2022 | 四君子汤合痛泻要方加减治疗腹泻型肠易激综合征的Meta分析 | 中国中医急症 |
| 13 | 吕晨辉;张艺;赵晶;厉祥媛;胡蓉;戴金锋 | 2022 | 中药联合四联疗法治疗幽门螺杆菌相关性胃炎的Meta分析 | 现代消化及介入诊疗 |
| 14 | 吕素;曹珊;李俊玲 | 2022 | 中西医结合治疗胃食管反流复发率的Meta分析 | 中医学报 |
| 15 | 吴俊松 | 2022 | 益气养阴法联合西药治疗IgA肾病的Meta分析及核心中药的网络药理学研究 | 湖北中医药大学（硕士学位论文） |
| 16 | 吴晓博;谭雨晴;田盼盼;陈恒文;李军 | 2021 | 补阳还五汤加减治疗慢性心衰临床疗效的系统评价及试验序贯分析 | 中国实验方剂学杂志 |
| 17 | 周锦涛 | 2021 | 中药联合常规方案补救治疗幽门螺杆菌的系统评价 | 陕西中医药大学（硕士学位论文） |
| 18 | 姚佳敏;唐梅文;夏琳超;覃瑶;许阳慧;李春林 | 2021 | 中医药对比匹维溴铵治疗腹泻型肠易激综合征疗效的Meta分析 | 环球中医药 |
| 19 | 孙伟茗 | 2021 | 中药辅助治疗H型高血压Meta分析 | 辽宁中医药大学（硕士学位论文） |
| 20 | 孙晓波;黄健;牛永军;白长川;石岩 | 2021 | 炙甘草汤治疗慢性心力衰竭的Meta分析 | 中医临床研究 |
| 21 | 孙童 | 2021 | 中药治疗尿酸性肾病的Meta分析 | 北京中医药大学（硕士学位论文） |
| 22 | 孟宪悦 | 2021 | 中医药对2型糖尿病患者胰岛功能影响的系统评价及用药规律分析 | 辽宁中医药大学（硕士学位论文） |
| 23 | 崔锦涛 | 2022 | 中药治疗特发性膜性肾病的Meta分析和网络药理学研究 | 湖北中医药大学（硕士学位论文） |
| 24 | 崔锦涛;陈幸萌;金劲松 | 2021 | 中药复方联合RAS抑制剂治疗IgA肾病的Meta分析 | 农垦医学 |
| 25 | 张令霖;连新福;陈昕;白永军;赵晶 | 2021 | 通窍活血汤联合西药治疗血管性痴呆临床疗效和安全性Meta分析 | 广州中医药大学学报 |
| 26 | 张恒恒;孙会卓;何文凤;孙千惠;李友林 | 2021 | 中药治疗慢性咳嗽的Meta分析 | 海南医学院学报 |
| 27 | 张慧敏 | 2021 | 排卵障碍性异常子宫出血证型分布、相关因素分析及中西医结合治疗AUB-O（气虚血瘀证）的Meta分析 | 成都中医药大学（硕士学位论文） |
| 28 | 张楚楚;刘莹;董燕;李斌;刘思鸿;李兵;张伟娜 | 2022 | 半夏泻心汤治疗浅表性胃炎临床随机对照试验的Meta分析 | 实用中医内科杂志 |
| 29 | 张洛闻;谢静 | 2022 | 中西医结合治疗小儿抽动症临床疗效的系统评价及Meta分析 | 中医临床研究 |
| 30 | 张津萌;张华东;赵亚男;石金杰;徐晓涵;刘宏潇 | 2021 | 中医药改善强直性脊柱炎疾病活动度的Meta分析 | 北京中医药 |
| 31 | 张瑞环;何旭;姚震;陈钧 | 2021 | 中药联合心境稳定剂治疗双相情感障碍的系统评价 | 中医药导报 |
| 32 | 张蕾；武志娟；张建伟 | 2022 | 中药复方治疗肾虚证不孕症临床疗效的meta分析 | 海南医学院学报 |
| 33 | 惠鑫蓉;孙化中;路霄健;张永志;魏峰明;韩红伟 | 2021 | 中药口服联合美沙拉嗪治疗溃疡性结肠炎临床疗效Meta分析 | 中医临床研究 |
| 34 | 方锐;周月;于明坤;陈凯飞;杨勇;刘萍;林宏远;柴玲;李思瑶;徐文峰;梅志刚;葛金文 | 2021 | 中药辨证联合降压药治疗高血压早期肾损害的系统评价与Meta分析 | 中草药 |
| 35 | 李云鹤 | 2021 | 类风湿关节炎继发骨质疏松症的文献计量分析及中药治疗荟萃分析 | 北京中医药大学（硕士学位论文） |
| 36 | 李吉旭;张林;李财;秦后伟 | 2022 | 半夏白术天麻汤治疗H型高血压Meta分析 | 中医临床研究 |
| 37 | 李心爱;李哲;商建伟;陈晓珩;祁烁;李会龙;丁治国 | 2022 | 中西医结合治疗桥本甲状腺炎疗效的meta分析 | 中国医药科学 |
| 38 | 李麟颖 | 2021 | 中西医结合治疗帕金森抑郁的meta分析 | 湖北中医药大学（硕士学位论文） |
| 39 | 杨婷 | 2022 | 中西医结合治疗多囊卵巢综合征性不孕症Meta分析 | 湖北中医药大学（硕士学位论文） |
| 40 | 杨翠娟; | 2021 | 温经汤加减治疗原发性痛经的Meta分析 | 世界最新医学信息文摘 |
| 41 | 杨萍;王海军;王少莲;乔明琦 | 2022 | 中医药治疗良性单纯性甲状腺结节临床疗效的Meta分析 | 湖北中医杂志 |
| 42 | 林宁;王嘉俊;廖伍萍;文莹 | 2022 | 中西医结合治疗桥本甲状腺炎疗效的Meta分析 | 西部中医药 |
| 43 | 林贤雷;林泽晨;钟亚珍;陆金华;林胜友 | 2021 | 中药治疗恶性肿瘤患者放射性肺损伤的疗效及安全性的Meta分析 | 中国现代医生 |
| 44 | 梁思灵;吴洋;邓亚萍;李世清;王兰 | 2021 | 中医药治疗高血压伴失眠疗效的系统评价 | 世界中西医结合杂志 |
| 45 | 楼蓉婕 | 2021 | 中医药治疗子宫腺肌病的meta分析 | 黑龙江中医药大学（硕士学位论文） |
| 46 | 毋洋洋 | 2021 | 中医药治疗功能性腹痛的meta分析及张骠教授治疗经验总结 | 南京中医药大学（硕士学位论文） |
| 47 | 毛天旭 | 2021 | 中药治疗月经过少的Meta分析及其证治规律 | 云南中医药大学（硕士学位论文） |
| 48 | 汪志伟;史孟华;宋太平;宋光瑞;刘全林 | 2021 | 中西医结合治疗溃疡性结肠炎疗效的meta分析 | 中国肛肠病杂志 |
| 49 | 王俊入 | 2021 | 近10年中药治疗早发性卵巢功能不全和卵巢早衰的Meta分析 | 云南中医药大学（硕士学位论文） |
| 50 | 王武浩 | 2021 | 近10年中西医结合治疗系统性红斑狼疮临床疗效及安全性的系统评价 | 广州中医药大学（硕士学位论文） |
| 51 | 王洪双 | 2021 | 中药治疗胃食管反流病的系统评价与Meta分析 | 河北中医学院（硕士学位论文） |
| 52 | 王蕾 | 2021 | 中医药治疗帕金森病睡眠障碍Meta分析 | 湖北中医药大学（硕士学位论文） |
| 53 | 王越悦;王凤荣;王帅 | 2021 | 中药联用ARBs治疗高血压病左心室肥厚疗效性与安全性系统评价 | 亚太传统医药 |
| 54 | 王辉;刘冬玲;韩祖成;惠振亮;袁捷;王瑾;骆凡;陈杰 | 2021 | 中医药治疗阿司匹林抵抗效果的Meta分析 | 临床医学研究与实践 |
| 55 | 童慧羲 | 2021 | 中医药治疗气滞血瘀型冠心病心绞痛的Meta分析 | 黑龙江中医药大学（硕士学位论文） |
| 56 | 罗惠;王宇飞;邓华兰 | 2021 | 中医药疗法治疗老年性功能性便秘的Meta分析及试验序贯分析 | 世界最新医学信息文摘 |
| 57 | 罗慜婧 | 2021 | 中药治疗慢性肾脏病湿热证的Meta分析与网络药理学研究 | 北京中医药大学（硕士学位论文） |
| 58 | 胡华卿 | 2021 | 健脾化痰补肾中药治疗多囊卵巢综合征的Meta分析和用药规律分析 | 广州中医药大学（硕士学位论文） |
| 59 | 胡梦奕;王永生;屠小龙;张婷素 | 2022 | 中药联合阿帕替尼治疗晚期胃癌疗效与安全性的Meta分析 | 浙江中西医结合杂志 |
| 60 | 董玉娟;林俊红;刘宴伟;周敏;欧阳炜 | 2021 | 含富碘中药复方治疗甲亢有效性与安全性的Meta分析 | 江西中医药 |
| 61 | 蔡晓月;陈善夫;李甜;赵英强 | 2022 | 中药治疗缓慢性心律失常的系统评价和Meta分析 | 光明中医 |
| 62 | 许丽璇;刘建璟;徐莹银;徐苏苏;李方霞;王丽丽;顾晓芸;魏文娟;蒋宽亮 | 2021 | 中药配方口服治疗干燥综合征疗效的Meta分析 | 湖南中医杂志 |
| 63 | 谢荣芳;刘丽婷;黄春华;饶旺福 | 2022 | 活血化瘀类中药治疗血管性头痛的Meta分析 | 江西中医药 |
| 64 | 贾元萍;王萱之;牛诗翔;邓妍童;,张洪春 | 2021 | 调补脾肾中药治疗慢性尿酸性肾病的系统评价 | 海南医学院学报 |
| 65 | 赖钰;许健;华艳朗;黎静仪;陈雪吟;刘云涛;王大伟 | 2021 | 温胆汤加减方治疗代谢综合征的疗效及安全性Meta分析 | 广州中医药大学学报 |
| 66 | 赵思郁;左韬;赵磊 | 2022 | 中西医结合治疗非增殖期糖尿病视网膜病变的荟萃分析 | 中国临床研究 |
| 67 | 逯文蕊;王凤荣 | 2021 | 中医药治疗经皮介入术(PCI)术后合并焦虑、抑郁的Meta分析 | 实用中医内科杂志 |
| 68 | 邹孟龙;黄晓燕;陈雅璐;宁芯 | 2021 | 四君子汤为基本方治疗溃疡性结肠炎的Meta分析 | 世界中医药 |
| 69 | 邹秀娟;郭玉芹;栾正丽;王云霞;吴春;李宏岩;杨加佳;丁媛 | 2021 | 中西医结合治疗风湿性多肌痛的疗效及安全性的Meta分析 | 中医临床研究 |
| 70 | 郑超;罗竖莹 | 2021 | 温胆汤加减治疗失眠症疗效及对PSQI评分影响的Meta分析 | 世界睡眠医学杂志 |
| 71 | 郭淳;黄楚冰;黄穗平;张北平 | 2021 | 中西医结合治疗克罗恩病临床疗效及安全性的系统评价与Meta分析 | 广州中医药大学学报 |
| 72 | 钱珍珍;张予晋;谢小丽;王军文 | 2022 | 中药汤剂联合HAART对HIV/AIDS患者免疫重建的系统评价 | 中国艾滋病性病 |
| 73 | 陈会君;董正;高媛;郝学东;陈星寒;客蕊 | 2022 | 半夏白术天麻汤联合降压药治疗原发性高血压有效性的Meta分析 | 中药药理与临床 |
| 74 | 陈晶晶 | 2021 | 中药治疗射血分数保留性心力衰竭的meta分析和作用机制研究 | 中国中医科学院（硕士学位论文） |
| 75 | 陈林娜 | 2022 | 中医药治疗子宫腺肌病的meta分析 | 湖北中医药大学（硕士学位论文） |
| 76 | 陈淼;史筱笑;安冬青 | 2021 | 桂枝汤类方治疗心系疾病的Meta分析 | 河南中医 |
| 77 | 陈颖;薛崇祥;杨德爽;姚睿祺;李玲玲;黄力 | 2022 | 基于Meta分析探讨益气活血类中药防治冠心病PCI术后再狭窄的优势 | 中西医结合心脑血管病杂志 |
| 78 | 章莉;徐泳;黄婧怡;何海浪;周贤梅 | 2021 | 射干麻黄汤化裁治疗小儿咳嗽变异性哮喘的Meta分析 | 中草药 |
| 79 | 马秀瑀 | 2021 | 半夏白术天麻汤及其加减治疗高血压病的Meta分析 | 黑龙江中医药大学（硕士学位论文） |
| 80 | 鲁强;马润芳;谢建辉;莫治密;何伟艺;赵华朗;李彩兰 | 2021 | 中药治疗幽门螺杆菌相关性胃炎的系统评价 | 时珍国医国药 |
| 81 | 龙佳 | 2021 | 口服中药治疗室性期前收缩随机对照试验的系统评价 | 北京中医药大学（硕士学位论文） |
| 82 | 黄淑霞;曹天雨;肖战说;殷海波 | 2022 | 中药联合硫酸羟氯喹治疗干燥综合征疗效和安全性的Meta分析 | 海南医学院学报 |

SR: systematic review.

Supplementary Table 2. Details of main analysis and subgroup analysis of binary and continuous treatment effects of Chinese herbal medicine: Chinese patent medicine (ref) vs. CHM decoctions in RCTs

| Subgroups | Binary treatment effects | | | | | Continuous treatment effects | | | | |
| --- | --- | --- | --- | --- | --- | --- | --- | --- | --- | --- |
|  | **Number of MAs (RCTs)** | **ROR (95%CI)** | I square | Tau square | **P** | **Number of MAs (RCTs)** | **dSMD (95%CI)** | I square | Tau square | **P** |
| Main analysis | 38 (585) | 1.13 [0.98, 1.30] | 0% | 0.00 | 0.09 | 12 (203) | -0.94 [-6.60, 4.71] | 30% | 26.19 | 0.74 |
| *Subgroup analysis by clinical conditions* | | | | | | | | | | |
| Diseases of the circulatory system | 6 (81) | 0.73 [0.47, 1.15] | 0% | 0.00 | 0.08 | 6 (83) | 3.17 [-3.53, 9.88] | 0% | 0.00 | 0.12 |
| Diseases of the genitourinary system | 7 (132) | 1.23 [0.90, 1.67] | 0% | 0.00 |  | 4 (81) | 0.88 [-12.81, 14.56] | 52% | 96.17 |  |
| Diseases of the digestive system | 7 (104) | 1.49 [1.12, 1.98] | 0% | 0.00 |  | 0 | NA | NA | NA |  |
| Endocrine, nutritional or metabolic diseases | 4 (60) | 1.06 [0.70, 1.60] | 0% | 0.00 |  | 1 (25) | -14.51 [-27.52, -1.50] | NA | NA |  |
| Other conditions | 14 (208) | 1.01 [0.78, 1.30] | 0% | 0.00 |  | 1 (14) | -3.68 [-18.20, 10.84] | NA | NA |  |
| *Subgroup analysis by nature of outcomes* | | | | | | | | | | |
| Subjective outcomes | 35 (535) | 1.12 [0.96, 1.31] | 0% | 0.00 | 0.74 | 1 (10) | 1.62 [-24.38, 27.62] | NA | NA | 0.85 |
| Objective outcomes | 3 (50) | 1.20 [0.81, 1.76] | 0% | 0.01 |  | 11 (193) | -0.99 [-6.92, 4.93] | 36% | 30.49 |  |
| *Subgroup analysis by funding support of SRs* | | | | | | | | | | |
| With funding | 21 (301) | 1.09 [0.89, 1.33] | 0% | 0.00 | 0.59 | 5 (68) | -1.16 [-8.56, 6.25] | 0% | 0.00 | 0.77 |
| Funding information not reported | 17 (284) | 1.18 [0.96, 1.45] | 0% | 0.00 |  | 7 (135) | 0.61 [-8.95, 10.17] | 56% | 87.97 |  |

ROR, risk of odds ratio; MAs, meta-analyses; RCT, randomized controlled trial; CI, confidence interval; SD, syndrome differentiation; SR, systematic review; dSMD, difference in standardized mean difference; CHM, Chinese herbal medicine. NA, not applicable. For binary treatment effects, a pooled ROR > 1 indicates that RCTs using CHM decoctions yielded larger binary treatment effects than RCTs using Chinese patent medicine; For continuous treatment effects, a pooled dSMD > 0 indicates that RCTs using CHM decoctions yield larger continuous treatment effects than RCTs using Chinese patent medicine.

**Supplementary Table 3. Details of sensitivity analysis of binary and continuous treatment effects, as well as adverse effects of Chinese herbal medicine: Chinese patent medicine (ref) vs. CHM decoctions in RCTs**

| Adjustment | Number of MAs (RCTs) | Binary treatment effects | | | | | Continuous treatment effects | | | | | | Adverse effects | | | |
| --- | --- | --- | --- | --- | --- | --- | --- | --- | --- | --- | --- | --- | --- | --- | --- | --- |
|  |  | **ROR**  **(95% CI)** | **I square** | **Tau square** | ***P*** | **Number of MAs (RCTs)** | | **dSMD**  **(95% CI)** | **I square** | **Tau square** | ***P*** | **Number of MAs (RCTs)** | **ROR**  **(95% CI)** | **I square** | **Tau square** | ***P*** |
| Unadjusted | 38 (585) | 1.13  [0.98, 1.30] | 0% | 0.00 | 0.09 | 12 (203) | | -0.94  [-6.60, 4.71] | 30% | 26.19 | 0.74 | 8 (141) | 0.80  [0.26, 2.47] | 75% | 1.87 | 0.70 |
| Adjusted for: | | | | | | | | | | | | | | | | |
| RCT sample size | 38 (585) | 1.03  [0.88, 1.22] | 0% | 0.00 | 0.68 | 12 (203) | | -2.22  [-4.75, 0.32] | 71% | 8.44 | 0.09 | 8 (141) | 0.97  [0.52, 1.81] | 35% | 0.14 | 0.92 |
| RCT funding | 38 (585) | 1.09  [0.93, 1.27] | 0% | 0.00 | 0.28 | 12 (203) | | -2.42  [-7.87, 3.04] | 21% | 19.78 | 0.39 | 8 (141) | 1.09  [0.64, 1.85] | 39% | ＜0.001 | 0.76 |
| RCT incorporating SD or not | 38 (585) | 1.16  [0.99, 1.35] | 0% | 0.00 | 0.06 | 12 (203) | | -0.26  [-6.68, 6.17] | 32% | 35.38 | 0.94 | 8 (141) | 0.59  [0.21, 1.67] | 71% | 1.49 | 0.32 |
| Random sequence generation | 38 (585) | 1.07  [0.92, 1.25] | 0% | 0.00 | 0.37 | 12 (203) | | -0.75  [-6.33, 4.84] | 31% | 20.09 | 0.79 | 8 (141) | 0.89  [0.26, 3.10] | 76% | 2.25 | 0.86 |
| Allocation concealment | 38 (585) | 1.13  [0.98, 1.32] | 0% | 0.00 | 0.10 | 12 (203) | | -0.65  [-7.47, 6.18] | 47% | 62.22 | 0.85 | 8 (141) | 0.84  [0.23, 3.13] | 81% | 2.62 | 0.80 |
| Blinding of participants and personnel | 38 (585) | 1.15  [1.00, 1.33] | 0% | 0.00 | 0.06 | 12 (203) | | -1.32  [-6.98, 4.35] | 28% | 22.73 | 0.65 | 8 (141) | 0.91  [0.29, 2.83] | 75% | 1.90 | 0.87 |
| Blinding of outcome assessment | 38 (585) | 1.15  [1.00, 1.33] | 0% | 0.00 | 0.05 | 12 (203) | | -0.94  [-6.60, 4.71] | 30% | 26.19 | 0.74 | 8 (141) | 0.83  [0.25, 2.74] | 76% | 2.15 | 0.76 |
| Incomplete outcome data | **38 (585)** | **1.18**  **[1.01, 1.37]** | **0%** | **0.00** | **0.03** | 12 (203) | | -1.97  [-8.22, 4.28] | 39% | 38.72 | 0.54 | 8 (141) | 0.79  [0.20, 3.17] | 74% | 2.59 | 0.74 |
| Selective reporting | 38 (585) | 1.11  [0.96, 1.29] | 0% | 0.00 | 0.14 | 12 (203) | | -1.05  [-6.61, 4.52] | 28% | 23.81 | 0.71 | 8 (141) | 0.81  [0.27, 2.42] | 73% | 1.76 | 0.70 |
| Adjusted for all covariates | 38 (585) | 0.97  [0.76, 1.24] | 0% | 0.00 | 0.98 | **12 (203)** | | **-3.72**  **[-6.91, -0.52]** | **65%** | **9.07** | **0.02** | 8 (141) | 0.73  [0.20, 2.62] | 0% | 0 | 0.73 |

ROR, risk of odds ratio; MAs, meta-analyses; RCT, randomized controlled trial; CI, confidence interval; SD, syndrome differentiation; dSMD, difference in standardized mean difference; CHM, Chinese herbal medicine. For binary treatment effects, a pooled ROR > 1 indicates that RCTs using CHM decoctions yielded larger binary treatment effects than RCTs using Chinese patent medicine. For continuous treatment effects, a pooled dSMD > 0 indicates that RCTs using CHM decoctions yield larger continuous treatment effects than RCTs using Chinese patent medicine. For adverse effects, a pooled ROR < 1 indicates that RCTs using CHM decoctions yielded less severe adverse effects than did RCTs using Chinese patent medicine.

Supplementary Table 4. Details of main analysis and subgroup analysis of binary and continuous treatment effects of Chinese herbal medicine: CHM Granules (ref) vs. CHM decoctions in RCTs

| Subgroups | Binary treatment effects | | | | | Continuous treatment effects | | | | |
| --- | --- | --- | --- | --- | --- | --- | --- | --- | --- | --- |
|  | **Number of MAs (RCTs)** | **ROR (95%CI)** | **I square** | **Tau square** | **P** | **Number of MAs (RCTs)** | **dSMD (95%CI)** | **I square** | **Tau square** | **P** |
| Main analysis | 38 (615) | 1.07 [0.91, 1.26] | 0% | 0 | 0.39 | 15 (259) | -0.81 [ -3.96, 2.33] | 6% | 0 | 0.61 |
| *Subgroup analysis by clinical conditions* | | | | | | | | | | |
| Diseases of the circulatory system | 3 (54) | 1.29 [0.76, 2.20] | 0% | 0 | 0.94 | 5 (83) | 0.91 [ -4.44, 6.26] | 0% | 0 | 0.3 |
| Diseases of the genitourinary system | 11 (192) | 1.15 [0.80, 1.64] | 26% | 0 |  | 5 (94) | -3.35 [ -8.68, 1.98] | 0% | 0 |  |
| Diseases of the digestive system | 11 (165) | 1.06 [0.70, 1.61] | 8% | 0 |  | NA | NA | NA | NA |  |
| Endocrine, nutritional or metabolic diseases | 2 (29) | 0.93 [0.52, 1.67] | 0% | 0 |  | 1 (25) | -9.26 [-21.25, 2.72] | NA | NA |  |
| Other conditions | 11 (175) | 1.07 [0.80, 1.45] | 0% | 0 |  | 4 (57) | 2.16 [ -5.68, 9.99] | 19% | 18 |  |
| *Subgroup analysis by nature of outcomes* | | | | | | | | | | |
| Subjective outcomes | 36 (586) | 1.06 [0.89, 1.26] | 0% | 0 | 0.55 | 2 (27) | -3.20 [-13.54, 7.14] | 0% | 0 | 0.64 |
| Objective outcomes | 2 (29) | 1.28 [0.70, 2.34] | 0% | 0 |  | 13 (232) | -0.59 [ -4.23, 3.05] | 17% | 5 |  |
| *Subgroup analysis by funding support of SRs* | | | | | | | | | | |
| With funding | 18 (259) | 1.01 [0.79, 1.29] | 0% | 0 | 0.46 | 7 (114) | 2.56 [ -2.57, 7.68] | 0% | 2 | 0.09 |
| Funding information not reported | 20 (356) | 1.15 [0.91, 1.44] | 0% | 0 |  | 8 (145) | -3.00 [ -7.02, 1.02] | 0% | 0 |  |

ROR, risk of odds ratio; MAs, meta-analyses; RCT, randomized controlled trial; CI, confidence interval; SD, syndrome differentiation; SR, systematic review; dSMD, difference in standardized mean difference; CHM, Chinese herbal medicine; NA, not applicable. For binary treatment effects, a pooled ROR > 1 indicates that RCTs using CHM decoctions yielded larger binary treatment effects than RCTs using CHM Granules. For continuous treatment effects, a pooled dSMD > 0 indicates that RCTs using CHM decoctions yield larger continuous treatment effects than RCTs using CHM Granules.

Supplementary Table 5. Details of sensitivity analysis of binary and continuous treatment effects, as well as adverse effects of Chinese herbal medicine: CHM Granules (ref) vs. CHM decoctions in RCTs

| **Adjustment** | **Binary treatment effects** | | | | | **Continuous treatment effects** | | | | | **Adverse effects** | | | | |
| --- | --- | --- | --- | --- | --- | --- | --- | --- | --- | --- | --- | --- | --- | --- | --- |
|  | **Number of MAs (RCTs)** | **ROR (95%CI)** | **I square** | **Tau square** | **P** | **Number of MAs (RCTs)** | **dSMD**  **(95%CI)** | **I square** | **Tau square** | **P** | **Number of MAs (RCTs)** | **ROR**  **(95%CI)** | **I square** | **Tau square** | **P** |
| Unadjusted | **38 (615)** | 1.07  [0.91, 1.26] | 0% | 0.00 | 0.39 | 15 (259) | -0.81  [-3.96, 2.33] | 6% | 0.10 | 0.61 | 3 (50) | 0.41  [0.10, 1.72] | 24% | 0.58 | 0.22 |
| Adjusted for: | | | | | | | | | | | | | | | |
| RCT sample size | 38 (615) | 1.13  [0.93, 1.36] | 0% | 0.00 | 0.21 | 15 (259) | -0.04  [-1.05, 0.97] | 0% | 0.00 | 0.94 | 3 (50) | 0.54  [0.10, 2.92] | 48% | 1.12 | 0.47 |
| RCT funding | 38 (615) | 1.05  [0.88, 1.24] | 0% | 0.00 | 0.61 | 15 (259) | -3.37  [-6.50, -0.23] | 0% | 4.18 | 0.04 | 3 (50) | 0.51  [0.10, 2.58] | 40% | 0.93 | 0.41 |
| RCT incorporating SD or not | 38 (615) | 1.07  [0.90, 1.27] | 0% | 0.00 | 0.43 | 15 (259) | -1.75  [-5.12, 1.62] | 0% | 0.00 | 0.31 | 3 (50) | 0.51  0.10, 2.54] | 47% | 0.99 | 0.41 |
| Random sequence generation | 38 (615) | 1.08  [0.91, 1.29] | 0% | 0.00 | 0.37 | 15 (259) | -0.86  [-4.03, 2.32] | 0% | 0.00 | 0.60 | 3 (50) | 0.75  [0.12, 4.88] | 70% | 1.66 | 0.77 |
| Allocation concealment | 38 (615) | 1.07  [0.90, 1.26] | 0% | 0.00 | 0.45 | 15 (259) | -0.33  [-3.47, 2.80] | 0% | 0.00 | 0.83 | 3 (50) | 0.65  [0.09, 4.62] | 75% | 1.93 | 0.67 |
| Blinding of participants and personnel | 38 (615) | 1.09  [0.92, 1.28] | 0% | 0.00 | 0.32 | 15 (259) | -2.28  [-5.63, 1.08] | 0% | 0.00 | 0.18 | 3 (50) | 0.39  [0.08, 1.91] | 38% | 0.86 | 0.25 |
| Blinding of outcome assessment | 38 (615) | 1.09  [0.93, 1.28] | 0% | 0.00 | 0.30 | 15 (259) | -2.28  [-5.63, 1.08] | 0% | 0.00 | 0.18 | 3 (50) | 0.41  [0.10, 1.71] | 22% | 0.56 | 0.22 |
| Incomplete outcome data | 38 (615) | 1.05  [0.89, 1.25] | 0% | 0.00 | 0.55 | 15 (259) | -2.06  [-5.49, 1.36] | 0% | 0.00 | 0.24 | 3 (50) | 0.42  [0.10, 1.70] | 21% | 0.54 | 0.22 |
| Selective reporting | 38 (615) | 1.09  [0.92, 1.28] | 0% | 0.00 | 0.32 | 15 (259) | -2.45  [-5.72, 0.82] | 0% | 0.00 | 0.14 | 3 (50) | 0.41  [0.10, 1.74] | 24% | 0.59 | 0.23 |
| Adjust for all covariates | 38 (615) | 1.25  [0.98,1.60] | 0% | <0.001 | 0.53 | 15 (259) | -0.07  [-1.38, 1.24] | 0% | 0 | 0.99 | 3 (50) | 0.70  [0.08, 5.78] | 68% | 2.11 | 0.05 |

ROR, risk of odds ratio; MAs, meta-analyses; RCT, randomized controlled trial; CI, confidence interval; SD, syndrome differentiation; dSMD, difference in standardized mean difference; CHM, Chinese herbal medicine. For binary treatment effects, a pooled ROR > 1 indicates that RCTs using CHM decoctions yielded larger binary treatment effects than RCTs using CHM Granules. For continuous treatment effects, a pooled dSMD > 0 indicates that RCTs using CHM decoctions yield larger continuous treatment effects than RCTs using CHM Granules. For adverse effects, a pooled ROR < 1 indicates that RCTs using CHM decoctions yielded less severe adverse effects than did RCTs using CHM Granules.

Supplementary Table 6. Details of sensitivity analysis of binary and adverse effects of Chinese herbal medicine: Chinese patent medicine (ref) vs. CHM Granules in RCTs

| Adjustment | Binary treatment effects | | | | | Adverse effects | | | | |
| --- | --- | --- | --- | --- | --- | --- | --- | --- | --- | --- |
|  | Number of MAs | ROR (95%CI) | I square | Tau square | P | Number of MAs | ROR (95%CI) | I square | Tau square | P |
| Unadjusted | 3 (59) | 0.90 [0.55, 1.47] | 3% | ＜0.001 | 0.67 | 1 (22) | 0.22 [0.05, 1.07] | NA | NA | NA |
| Adjusted for: | | | | | | | | | | |
| RCT sample size | 3 (59) | 1.06 [0.56, 1.99] | 0% | 0 | 0.86 | 1 (22) | 0.33 [0.06, 1.96] | NA | NA | NA |
| RCT funding | 3 (59) | 0.85 [0.51, 1.41] | 0% | 0 | 0.53 | 1 (22) | 0.27 [0.04, 1.65] | NA | NA | NA |
| RCT incorporating SD or not | 3 (59) | 0.85 [0.50, 1.43] | 7% | 0.011 | 0.54 | 1 (22) | 0.22 [0.05, 1.07] | NA | NA | NA |
| Random sequence generation | 3 (59) | 0.90 [0.55, 1.48] | 2% | ＜0.001 | 0.68 | 1 (22) | 0.22 [0.05, 1.07] | NA | NA | NA |
| Allocation concealment | 3 (59) | 0.88 [0.54, 1.45] | 0% | ＜0.001 | 0.62 | 1 (22) | 0.25 [0.05, 1.26] | NA | NA | NA |
| Blinding of participants and personnel | 3 (59) | 0.87 [0.53, 1.43] | 0% | 0 | 0.58 | 1 (22) | 0.27 [0.06, 1.31] | NA | NA | NA |
| Blinding of outcome assessment | 3 (59) | 0.88 [0.54, 1.45] | 0% | ＜0.001 | 0.62 | 1 (22) | 0.25 [0.05, 1.26] | NA | NA | NA |
| Incomplete outcome data | 3 (59) | 0.90 [0.55, 1.47] | 4% | ＜0.001 | 0.67 | 1 (22) | 0.23 [0.04, 1.18] | NA | NA | NA |
| Selective reporting | 3 (59) | 0.89 [0.54, 1.47] | 0% | ＜0.001 | 0.65 | 1 (22) | 0.22 [0.04, 1.13] | NA | NA | NA |
| Adjusted for all covariates | 3 (59) | 0.88 [0.33, 2.29] | 0% | 0.158 | 0.45 | 1 (22) | 0.17 [0.00,7.21] | NA | NA | NA |

ROR, risk of odds ratio; MAs, meta-analyses; RCT, randomized controlled trial; CI, confidence interval; SD, syndrome differentiation; CHM, Chinese herbal medicine; NA, not applicable. For binary treatment effects, a pooled ROR < 1 indicates that RCTs using Chinese patent medicine yielded larger binary treatment effects than RCTs using CHM Granules. For adverse effects, a pooled ROR < 1 indicates that RCTs using CHM patent medicine yielded more severe adverse effects than did RCTs using CHM Granules.

Supplementary Table 7. Results of Egger’s test for all outcomes with over 10 studies

| **Comparison** | **Outcome** | **Adjusted for covariates** | **Test result** |
| --- | --- | --- | --- |
| CHM Granules vs. CHM decoction | Binary treatment effects | Main analysis (No adjust) | t = 2.02, p-value = 0.051 |
| CHM Granules vs. CHM decoction | Binary treatment effects | Adjusted for all covariates | t = 0.65, p-value = 0.517 |
| CHM Granules vs. CHM decoction | Continuous treatment effects | Main analysis (No adjust) | t= -0.59, p-value= 0.565 |
| CHM Granules vs. CHM decoction | Continuous treatment effects | Adjusted for all covariates | t= -0.35, p-value=0.731 |
| Chinese patent medicine vs. CHM decoction | Binary treatment effects | Main analysis (No adjust) | t= -1.52, p-value=0.136 |
| Chinese patent medicine vs. CHM decoction | Binary treatment effects | Adjusted for all covariate | t= 0.08, p-value=0.939 |
| Chinese patent medicine vs. CHM decoction | Continuous treatment effects | Main analysis (No adjust) | t=1.46, p-value=0.175 |
| Chinese patent medicine vs. CHM decoction | Continuous treatment effects | Adjusted for all covariate | t= -1.08, p-value=0.307 |

CHM, Chinese herbal medicine.

Supplementary Material C. Funnel plots for all outcomes with over 10 studies.


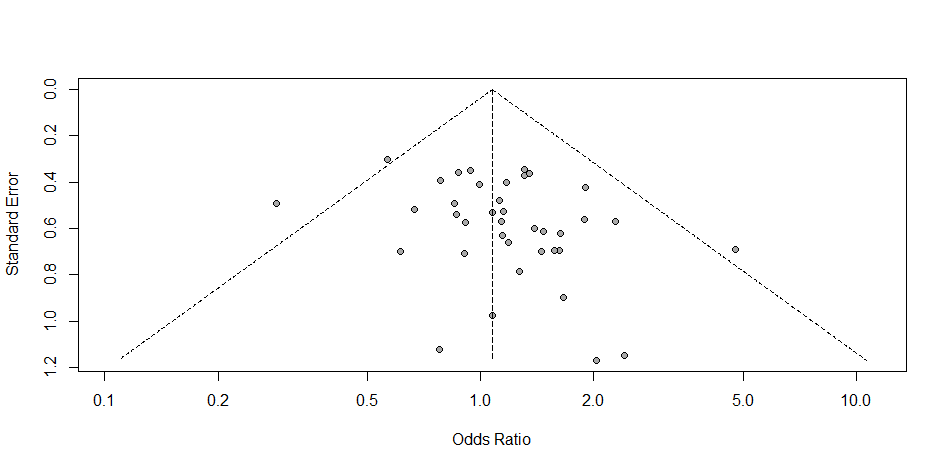
Supplementary Figure 1. Funnel plot for main analysis of binary treatment effects: CHM Granules *vs.* CHM decoction. CHM, Chinese herbal medicine.


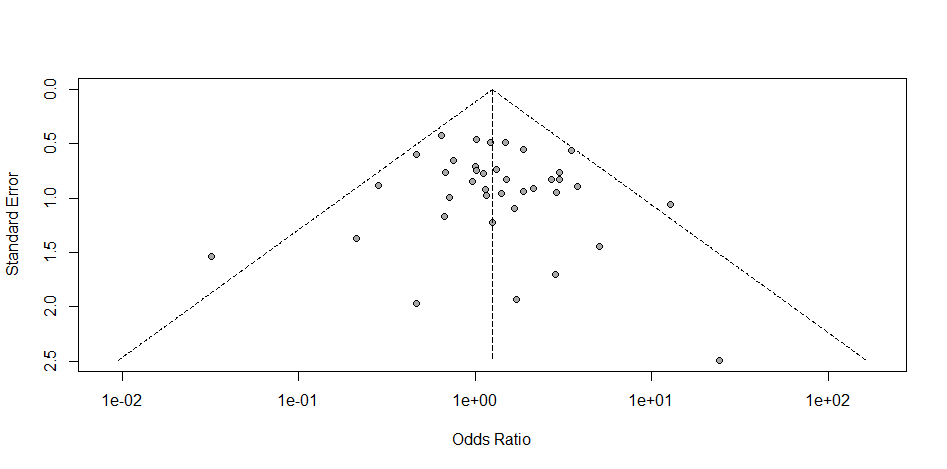


Supplementary Figure 2. Funnel plot for sensitivity analysis of binary treatment effects adjusted for all potential covariates: CHM Granules *vs.* CHM decoction. CHM, Chinese herbal medicine.


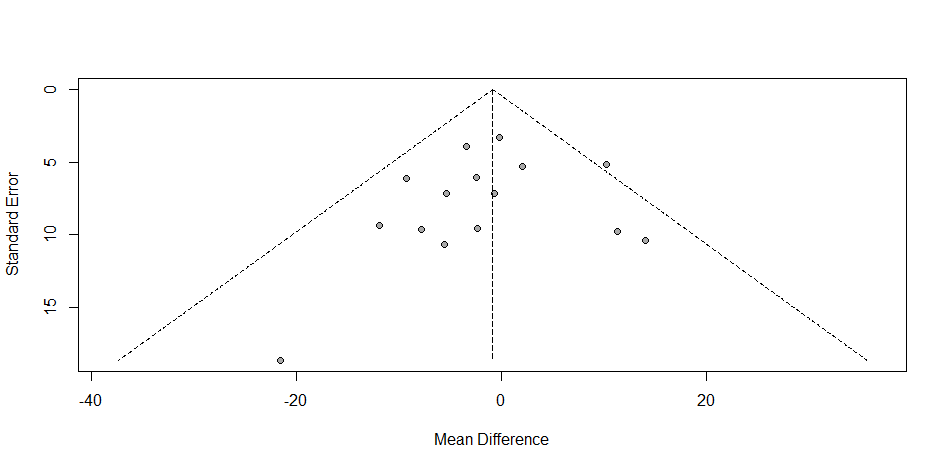


Supplementary Figure 3. Funnel plot for main analysis of continuous treatment effects: CHM Granules *vs.* CHM decoction. CHM, Chinese herbal medicine.


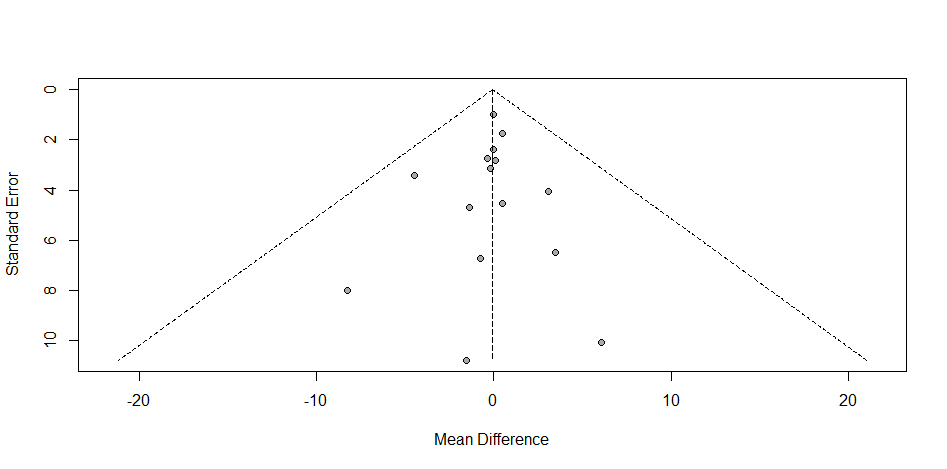


Supplementary Figure 4. Funnel plot for sensitivity analysis of continuous treatment effects adjusted for all potential covariates: CHM Granules *vs.* CHM decoction. CHM, Chinese herbal medicine.


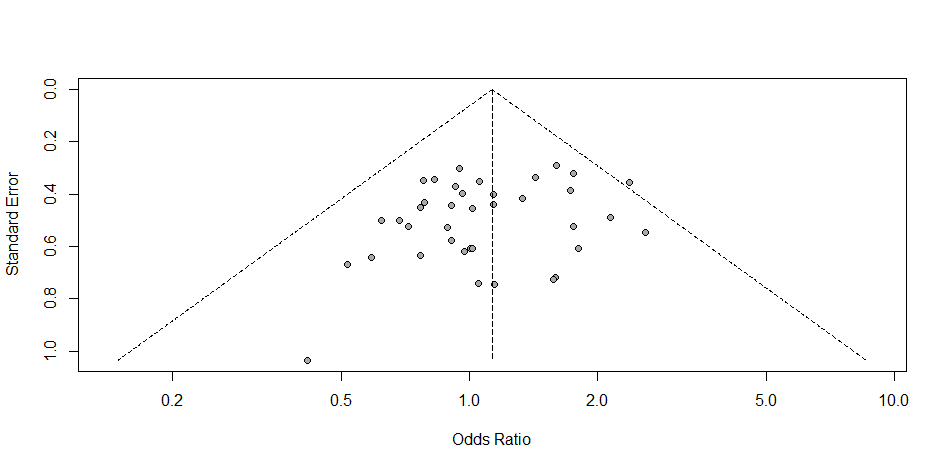


Supplementary Figure 5. Funnel plot for main analysis of binary treatment effects: Chinese patent medicine *vs.* CHM decoction. CHM, Chinese herbal medicine.


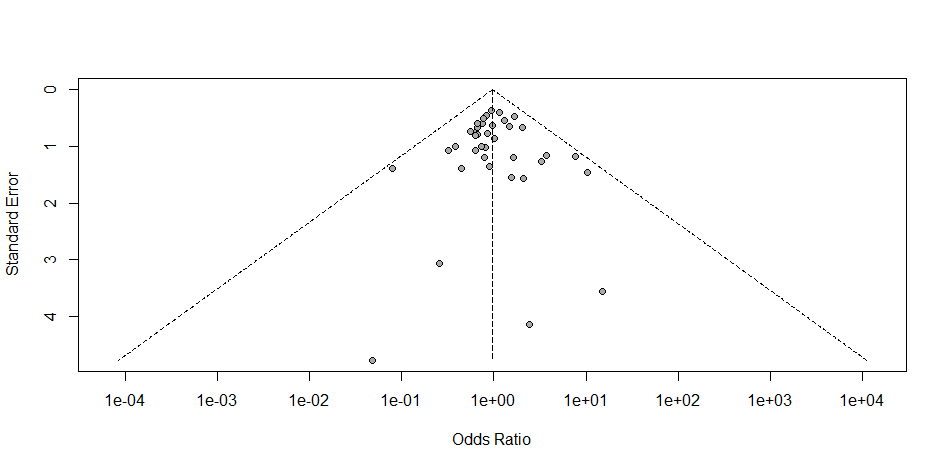


Supplementary Figure 6. Funnel plot for sensitivity analysis of binary treatment effects adjusted for all potential covariates: Chinese patent medicine *vs.* CHM decoction. CHM, Chinese herbal medicine.


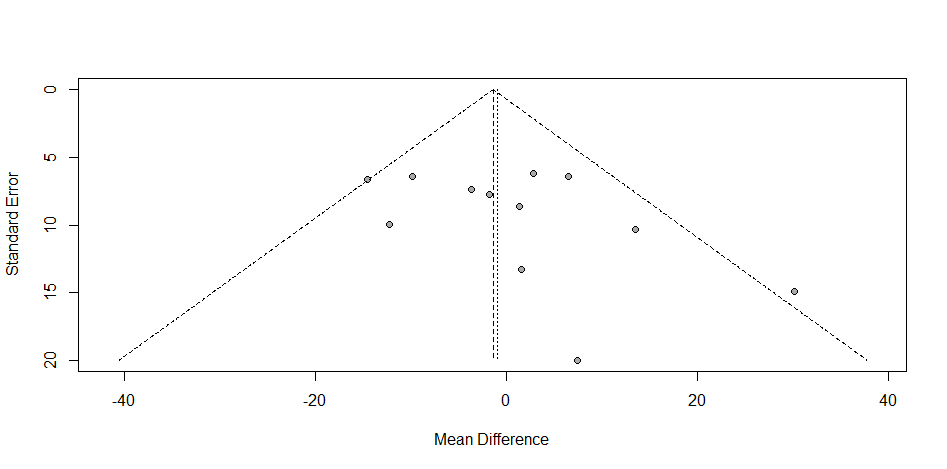


Supplementary Figure 7. Funnel plot for main analysis of continuous treatment effects: Chinese patent medicine *vs.* CHM decoction. CHM, Chinese herbal medicine.


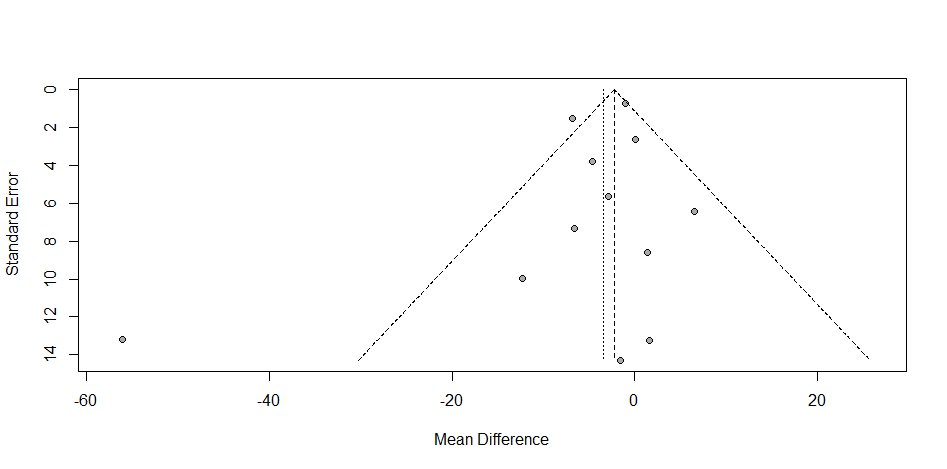


Supplementary Figure 8. Funnel plot for sensitivity analysis of continuous treatment effects adjusted for all potential covariates: Chinese patent medicine *vs.* CHM decoction. CHM, Chinese herbal medicine.
